# Ethnic density and other neighbourhood associations for mortality in severe mental illness: a retrospective cohort study with multi-level analysis from an urbanised and ethnically diverse location in the UK

**DOI:** 10.1016/S2215-0366(19)30126-9

**Published:** 2019-06

**Authors:** Jayati Das-Munshi, Peter Schofield, Vishal Bhavsar, Chin-Kuo Chang, Michael E Dewey, Craig Morgan, Robert Stewart, Graham Thornicroft, Martin J Prince

**Affiliations:** a Department of Psychological Medicine, Institute of Psychiatry, Psychology and Neurosciences, King's College London, London, UK; b Department of Health Services and Population Research, Institute of Psychiatry, Psychology and Neurosciences, King's College London, London, UK; c Department of Population Health Sciences, Faculty of Life Sciences and Medicine, King's College London, London, UK; d South London and Maudsley National Health Service Foundation Trust, London, UK; e Department of Health and Welfare, University of Taipei, Taipei City, Taiwan

## Abstract

**Background:**

Neighbourhood social context might play a role in modifying mortality outcomes in severe mental illness, but has received little attention to date. Therefore, we aimed to assess in an ethnically diverse and urban location the association of neighbourhood-level characteristics and individual-level factors for all-cause, natural-cause, and unnatural-cause mortality in those with severe mental illness.

**Methods:**

We did a retrospective cohort study using a case-registry from a large secondary mental health-care Trust in an ethnically diverse and urban location in south London, UK. Linked data for deaths and areas of residence were identified from the case-registry. We included all individuals aged 15 years or more at the time of diagnosis for a severe mental illness from Jan 1, 2007, to Dec 31, 2014. We used individual-level information in our analyses, such as gender, marital status, and the presence of current or previous substance use disorders. We assessed neighbourhood or area-level indicators at the Lower Super Output Area level. Association of neighbourhood-level characteristics, which included the interaction between ethnicity and own ethnic density, deprivation, urbanicity, and social fragmentation, alongside individual-level factors for all-cause, natural-cause, and unnatural-cause mortality in those with severe mental illness was assessed.

**Findings:**

A total of 18 201 individuals were included in this cohort for analyses, with a median follow-up of 6·36 years. There were 1767 (9·7%) deaths from all causes, 1417 (7·8%) from natural causes, and 192 (1·1%) from unnatural causes. In the least ethnically dense areas, the adjusted rate ratio (aRR) for all-cause mortality in ethnic minority groups with severe mental illness compared with white British people with severe mental illness were similar (aRR 0·96, 95% CI 0·71–1·29); however in the highest ethnic density areas, ethnic minority groups with severe mental illness had a lower risk of death (aRR 0·52, 95% CI 0·38–0·71; p<0·0001), with similar trends for natural-cause mortality (p=0·071 for statistical interaction). In the cohort with severe mental illness, residency in deprived, urban, and socially fragmented neighbourhoods was not associated with higher mortality rates. Compared with the general population, age-standardised and gender-standardised mortality ratios were elevated in the cohort with severe mental illness across all neighbourhood-level characteristics assessed.

**Interpretation:**

For ethnic minority groups with severe mental illness, residency in areas of higher own-group ethnic density is associated with lower mortality compared to white British groups with severe mental illness.

**Funding:**

Health Foundation, National Institute for Health Research, EU Seventh Framework, and National Institute of Mental Health.

## Introduction

Across international settings, people with severe mental illness, such as schizophrenia and bipolar disorder, have a markedly elevated risk of death.^1^ Mortality in these populations is mostly from preventable physical causes, and the gap in life expectancy is thought to be increasing over time.^1^ Approaches to address this issue have tended to focus on individual-level risks for physical health,^2^ despite a growing acknowledgement that such approaches miss opportunities at the population level for intervention.^3^

Almost 20 years ago WHO coordinated a series of studies on the long-term outcomes of schizophrenia, using identical epidemiological case-finding methods for schizophrenia across international centres.^4^, ^5^, ^6^, ^7^ The investigators noted that although the absolute risk of mortality in severe mental illness was increased compared with national populations across all surveyed contexts, standardised mortality ratios were most increased in industrialised centres compared with non-industrialised centres.^4^, ^5^, ^6^, ^7^ The notion that outcomes in severe mental illness, including mortality, might be more benign in less-developed countries has been severely critiqued.^8^ However, it remains a possibility that strong social factors in the environment, beyond traditional risk factors measured at the individual level, might modify the excess risk of mortality in people living with severe mental illness. The WHO study investigators postulated that socioenvironmental factors, such as family involvement, community solidarity, cohesive neighbourhoods, and other factors mitigating against social isolation, potentially have an important role in outcomes such as mortality.^5^, ^6^, ^7^, ^9^ In particular, a more recent finding has been that for certain ethnic minority groups with severe mental illness, mortality outcomes might be lower than in reference groups with severe mental illness.^10^ For ethnic minority groups in the general population, residency in areas of higher own ethnic density might confer health benefits through enhanced social support and buffering against social isolation and exclusion for marginalised groups.^11^, ^12^ No studies to date have assessed either this hypothesis or the possibility that other neighbourhood or group-level factors have a role in mitigating against elevated mortality in people with severe mental illness.

**Research in context**

**Evidence before this study**

People with severe mental illness have major reductions in life-expectancy and a markedly elevated risk of death compared with the general population across all international contexts surveyed. To date, most previous studies have focused on risk factors at the individual level (such as tobacco use, diet, and physical activity) for mortality; however, there are growing calls to consider the role of broader social and contextual determinants.

We searched MEDLINE for studies examining area-level factors associated with mortality in people with mental illness, from inception to Feb 6, 2019, with no language restrictions. We used the MeSH terms, “Mortality” and “Mental illness”, and keywords including “residential characteristics”, “neighbourhood characteristics”, “multilevel analysis”, “ethnic density”, “social fragmentation”, “urban”, and “deprivation”. We used a previous systematic review to inform the search terms used in our searches (appendix). We also manually searched the reference list of another recently published systematic review. For inclusion, eligible studies had to have relevant area-level indicators and associations with mortality presented, include populations consisting of people with severe mental illness, and have an effect size for the association of the area-level indicator with mortality outcomes. We excluded ecological studies. A total of 1281 studies were identified, which were screened by abstract or full text for possible inclusion. A total of eight studies were identified as relevant. Six focused on all-cause mortality of which one study also included deaths from natural and unnatural causes, and the other two studies focused on suicide. The studies also assessed area-level exposures across international settings, which included Ethiopia (two studies), China (one study), Taiwan (one study), UK and devolved countries (two studies), and Canada (two studies).

Across all studies, irrespective of area-level associations, people with severe mental illness had increased mortality compared with reference groups without severe mental illness. Associations with area-level indicators and mortality outcomes followed differing patterns, dependent on context. There were mixed findings across studies examining the effect of urbanicity on mortality outcomes in severe mental illness. This included one study suggesting an increased risk of death in urban compared with rural areas (from Ethiopia), one study indicating an increased risk in rural compared with urban areas (from China), and two studies suggesting no association (from Canada and Ethiopia). Four out of five retrieved studies supported residency in more deprived areas being associated with elevated mortality in people with severe mental illness. However, in one study from a nationally representative sample from the UK, the investigators reported that people with severe mental illness resident in more deprived areas had a lower risk of suicide than people resident in less deprived areas. No studies were identified that assessed the association of ethnic density or social fragmentation with mortality in severe mental illness.

**Added value of this study**

This study is the first to systematically assess a range of neighbourhood-level exposures and, specifically, ethnic density, with mortality in severe mental illness. The study was situated in a geographically defined, ethnically diverse, urban location in the UK. We found strong support for the observation that, for certain ethnic minority groups with severe mental illness, mortality risks (especially for deaths from all causes and natural causes) are lower in areas of higher own-group density relative to white British people with severe mental illness. Other area-level indicators (social fragmentation, urbanicity, and area-level deprivation) as assessed in this study were not associated with excess mortality in severe mental illness. Our findings build on a previous analysis that had shown that risks of all-cause, natural-cause, and unnatural-cause mortality were lower in most UK ethnic minority groups with severe mental illness compared with a white British reference group with severe mental illness. The present study suggests that the ethnic density of the area where ethnic minority groups with severe mental illness reside might have an important role in modifying mortality risks. Residency in areas of higher own-group density might buffer against social isolation or might be associated with exposure to stronger health-protective social norms, which might ultimately protect against premature mortality in severe mental illness. Our findings challenge approaches that focus purely on individual-level risk factors.

**Implications of all the available evidence**

Premature mortality in people with mental illness might reflect a complex array of both individual and contextual or neighbourhood-level influences. Future studies must account for both individual-level and group-level sources of variation in mortality in these populations. A clearer understanding of the sources of variation could be used to guide the development of interventions to address premature mortality in people with severe mental illness and should be explored in the future.

To address this gap in the literature, we aimed to assess in an ethnically diverse and urban location the association of neighbourhood-level characteristics alongside individual-level factors, with all-cause, natural-cause, and unnatural-cause mortality in people with severe mental illness. Additionally, we assessed whether an interaction between area-level ethnic density and individual-level ethnicity exists. We hypothesised that individuals of an ethnic minority background with severe mental illness would have lower mortality risks when living in areas of higher ethnic density and that mortality would be increased in all people with severe mental illness living in neighbourhoods that were more deprived, urbanised, and socially fragmented.

## Methods

### Study design and participants

We did a retrospective cohort study in an ethnically diverse and urban location in south London, UK. South London and Maudsley National Health Service (NHS) Trust is one of Europe's largest secondary mental health-care providers.^13^ The Trust provides comprehensive secondary mental health care to a catchment area of approximately 1·36 million people in an ethnically diverse location in south London, which includes the London Boroughs of Lambeth, Lewisham, Croydon, and Southwark. According to the 2011 Office for National Statistics' rural-urban classification system, these boroughs are considered urban with major conurbation areas, defined as built-up areas with resident populations of more than 10 000 people. The London Borough of Croydon also contains less urban areas towards the south of the borough.

Since 2006, South London and Maudsley NHS Trust has operated fully electronic health records. The Clinical Record Interactive Search (CRIS) system, established in 2008, is an ethically approved electronic health records interface system that allows researchers to access de-identified electronic health records from this Trust for research.^13^, ^14^ All clinicians and mental health teams are required to assign psychiatric diagnoses according to ICD-10 to patients who make contact with the mental health Trust. Individuals with ICD-10 diagnoses of severe mental illness, specifically schizophrenia-spectrum disorders (F2*) and bipolar disorders (F30 and F31), were identified through searches of CRIS-structured fields, supplemented by a Natural Language Processing application developed with Generalised Architecture for Text Engineering, which extracts diagnostic statements from the free text of case notes and clinical correspondence.^13^ A 2018 audit of the performance of the Natural Language Processing algorithm that was used to determine clinical diagnoses from the free-text fields, which supplemented the structured field diagnoses entered by clinicians, found that for severe mental illness as a whole recall (ie, sensitivity) was 0·43 and precision (positive predictive value) was 0·95. For schizophrenia this sensitivity was 0·63 and positive predictive value was 0·96. These figures reflect performance of the application at annotation level; we would expect sensitivity to be higher at the patient level since there would be repeat annotations, which has not yet been assessed.

We obtained permission to do secondary analysis of the CRIS system from the Oxfordshire Research Ethics Committee C (reference 18/SC/0372). Additionally, we obtained separate approvals to examine linked mortality data with approved researcher status from the UK Health and Social Care Information Centre.

All individuals aged 15 years or more at the time of diagnosis after contact with any of the services (inpatient, outpatient, and accident and emergency contacts) from South London and Maudsley NHS Trust were included in the cohort. Individuals with comorbid dementia diagnoses before the diagnosis of severe mental illness were excluded. The observation period for this study was from Jan 1, 2007, to Dec 31, 2014, with individuals entering the cohort at the date of their severe mental illness diagnosis.

The protocol for this study is registered with Open Science Framework and is available online.

### Measures

We used individual-level information in our analyses, which included gender, marital status (married or cohabiting, divorced, separated, widowed, and single), and the presence of current or previous substance use disorders (alcohol and drug use). We also used birth date to derive age, which was handled in the analyses as a time-changing variable. We classified self-ascribed ethnicity according to the UK Office for National Statistics criteria for the main ethnic groups: white British, black Caribbean, black African, south Asian, and Irish groups, which were also aggregated into a binary white British and ethnic minority variable. We classified ICD-10 F30 and F31 codes as affective diagnoses and ICD-10 code F2* as non-affective disorders. Previous or current substance use disorder diagnoses were determined by the presence of an ICD-10 code for F10–F19 (mental and behavioural disorders due to psychoactive substance use), noted at some point in the clinical record.

We assessed neighbourhood or area-level indicators at the Lower Super Output Area (LSOA) level. LSOAs are national administrative areas that have a mean population of 1614 individuals.^15^ Area-level measures were calculated for the LSOAs based on individuals' addresses at the time of their first recorded severe mental illness diagnosis. We used the Index for Multiple Deprivation from 2010 to assess multiple deprivation across seven domains: income, employment, health and disability, education, housing, crime, and living environment.^16^ We mapped the Index for Multiple Deprivation to LSOA boundaries from 2011 (ie, the midpoint of the observation window).

We determined all other area-level indicators at the LSOA using publicly available data from the UK Office for National Statistics. Urbanicity at the LSOA level was derived from the 2011 Census data by calculating the number of people per hectare of land.^17^ We derived an indicator for social fragmentation based on the theoretically informed approaches taken by previous investigators,^18^, ^19^, ^20^ using 2011 census data at the LSOA level. Census variables for numbers of privately rented households, single-person households, and unmarried individuals or individuals not in cohabiting relationships at the LSOA level, and mobility in the previous year (ie, numbers not resident at the same address 1 year previously) at the Middle Super Output Area level were used and then standardised, generating Z scores. Z scores for each of these variables were then added together, resulting in a social fragmentation variable, which was divided at the quartile, leading to four categories, with the upper fourth further divided at the 90th percentile, because of the skew of the variable.^19^ The resultant social fragmentation variable comprised five categories ranging from the least to the most socially fragmented areas.

Own-group ethnic density for each of the LSOAs was determined as the percentage of the total population accounted for by each of the minority ethnic groups resident in the area at the time of the 2011 Census and derived for black Caribbean, black African, south Asian, and Irish groups. An overall ethnic density variable was also derived, which was the proportion of the total population in each area who were ethnic minority residents. The ethnic density variable was retained as a continuous variable across all regression models. In order to estimate standardised mortality ratios in the sample, we also derived a categorical ethnic density variable, whereby the continuous variable was divided into ten equal groups, with standardised mortality ratios estimated for the lowest and highest groups.

Details of deaths for patients who had received care from the South London and Maudsley NHS Trust were provided by the UK Office of National Statistics and linked to health records using unique patient identifiers. The linkage meant that death certificate information with date and cause of death according to ICD-10 was available for all cohort members who died during the study observation period.^21^ Deaths were classified as from all causes (A00-R99; U00-Y89), natural causes (A00-Q99), and unnatural causes, which included deaths from suicide and external causes (U509, V01-Y89). Deaths not otherwise classified (R00-R99) were included in analyses of all-cause mortality but not analysed separately.

### Statistical analysis

We derived standardised mortality ratios through indirect age-standardisation and gender-standardisation to the resident population and deaths in England and Wales, in 2011. Age was determined at the midpoint of the observation period (Jan 1, 2011) or at the diagnosis date of the mental disorder if this diagnosis occurred after Jan 1, 2011. Age was categorised into 10-year bands corresponding to the reference population's age groups. As the cohort was followed-up for varying periods, depending on diagnosis dates within the 8-year observation window, we derived weights taking the mean observation periods contributed by cohort members within each age and gender band. Weights were then multiplied by the number of deaths recorded in each corresponding age and gender band for the standard population, which had been observed for 1 year only, to generate the expected numbers of death as the denominators of standardised mortality ratios for the cohort. Standardised mortality ratios were then calculated for all-cause, natural-cause, and unnatural-cause mortality for each of the area-level indicators (deprivation, urbanicity, social fragmentation, and ethnic density) at the highest and lowest levels.

Individuals were followed-up from date of their diagnosis until death, emigration, or the end of the observation window on Dec 31, 2014. For all-cause mortality, multi-level Poisson regression using the mepoisson command in Stata were used specifying individuals nested within LSOAs. A cross-level interaction was fitted between self-ascribed ethnicity (at the individual level) and own ethnic density (at the area level) for all models. Initially, this was for a binary ethnicity variable (white British [reference], all ethnic minority groups [exposure]), but was then repeated for each interaction (ethnicity × own ethnic density). For the ethnic minority groups and own ethnic density analyses, we present the stratum-specific estimates for the ethnic minority group in question. Lexis expansion was used to model age as a time-changing variable, which was grouped into three bands (age 15–44, 45–64, and ≥65 years).

To model deaths from natural and unnatural causes, a competing-risks regression was fitted, using the stcrreg command in Stata.^22^ These approaches estimate sub-distribution hazard ratios (HRs), with 95% CIs, for deaths from a specified cause, while taking into account the competing risk of death from other causes occurring during the observation period.^22^ Using this approach, models assessing the association of risk factors with natural-cause mortality were assessed, specifying unnatural-cause mortality as the competing risk and vice versa. We used robust standard errors to adjust for clustering at LSOA level across these models and used Wald tests to assess for associations and interactions. We also used this approach to run a post-hoc sensitivity analysis, testing our models against the possibility that migrant groups with poorer health might be more likely to migrate back to their country of origin before death, leading to a numerator-denominator mismatch and a false impression of healthy migrant effects.^23^ This is relevant as a large proportion of the ethnic minority groups in the cohort might have been first-generation migrant groups. We specified date of emigration out of the cohort as a competing risk against all-cause mortality in this sensitivity analysis.^10^ For each continuous variable (area-level deprivation, urbanicity, social fragmentation, ethnic density, and age), a linear association for the variable with outcome was assessed compared with a categorical or quadratic association, using likelihood ratio tests (in multi-level models for all-cause mortality) or Wald tests (in competing risk regression models for natural and unnatural mortality). A quadratic relationship between age and outcomes and a linear relationship between area-level variables and outcomes were noted across all models and therefore used in this form in the final analyses. We used Stata/SE (version 13.1) for data analyses.

## Results

A total of 18 201 individuals contributed 122 731 person-years to the cohort for analyses, with a median follow-up time of 6·36 years. There were 1767 deaths from all causes, including 1417 deaths due to natural causes, 192 deaths due to unnatural causes, and 158 deaths from unknown causes, corresponding to an overall all-cause crude mortality rate of 14·4 deaths per 1000 person-years (95% CI 13·7–15·1; table 1).

**Table 1:** Demographic composition, deaths, and crude mortality rates in the sample

			**Total sample**	**All-cause mortality**		**Natural cause mortality**		**Unnatural cause mortality**	
				All-cause deaths	Crude rate (95% CI)	Natural-cause deaths	Crude rate (95% CI)	Unnatural-cause deaths	Crude rate (95% CI)
Total			18 201 (100%)	1767 (9·7%)	14·4 (13·7–15·1)	1417 (7·8%)	11·5 (11·0–12·2)	192 (1·1%)	1·6 (1·4–1·8)
Area level indicators									
	Index of Multiple Deprivation (rank score)								
		4 (most deprived)	3667 (20·1%)	377 (10·3%)	14·4 (13·0–16·0)	305 (8·3%)	11·7 (10·4–13·1)	33 (0·9%)	1·3 (0·9–1·8)
		4276	3646 (20·0%)	342 (9·4%)	13·3 (12·0–14·8)	273 (7·5%)	10·6 (9·4–12·0)	35 (1·0%)	1·4 (1·0–1·9)
		6144	3629 (19·9%)	313 (8·6%)	12·5 (11·2–14·0)	247 (6·8%)	9·9 (8·7–11·2)	37 (1·0%)	1·5 (1·1–2·0)
		8747	3673 (20·2%)	371 (10·1%)	14·9 (13·5–16·5)	297 (8·1%)	12·0 (10·7–13·4)	39 (1·1%)	1·6 (1·1–2·1)
		12 859 (least deprived)	3586 (19·7%)	364 (10·2%)	17·3 (15·6–19·2)	295 (8·2%)	14·0 (12·5–15·7)	48 (1·3%)	2·3 (1·7–3·0)
	Urbanicity (persons per hectare)								
		0·1 (least populous)	3613 (19·9%)	377 (10·4%)	17·5 (15·8–19·4)	301 (8·3%)	14·0 (12·5–15·7)	42 (1·2%)	2·0 (1·4–2·6)
		55·3	3762 (20·7%)	370 (9·8%)	15·0 (13·5–16·6)	282 (7·5%)	11·4 (10·2–12·8)	49 (1·3%)	2·0 (1·5–2·6)
		88·6	3667 (20·1%)	347 (9·5%)	13·5 (12·1–14·9)	275 (7·5%)	10·7 (9·5–12·0)	41 (1·1%)	1·6 (1·2–2·2)
		111·4	3652 (20·1%)	374 (10·2%)	14·7 (13·3–16·2)	315 (8·6%)	12·4 (11·1–13·8)	29 (0·8%)	1·1 (0·8–1·6)
		145·0 (most populous)	3507 (19·3%)	299 (8·5%)	11·8 (10·6–13·3)	244 (7·0%)	9·7 (8·5–11·0)	31 (0·9%)	1·2 (0·9–1·7)
	Social fragmentation								
		−7·2 (least fragmented)	4669 (25·7%)	451 (9·7%)	15·8 (14·4–17·3)	364 (7·8%)	12·7 (11·5–14·1)	51 (1·1%)	1·8 (1·4–2·3)
		−2·4	4593 (25·2%)	477 (10·4%)	15·0 (13·7–16·4)	390 (8·5%)	12·3 (11·1–13·6)	45 (1·0%)	1·4 (1·1–1·9)
		−0·2	4534 (24·9%)	469 (10·3%)	14·7 (13·4–16·1)	366 (8·1%)	11·5 (10·4–12·7)	52 (1·1%)	1·6 (1·2–2·1)
		2·0	2686 (14·8%)	227 (8·5%)	12·2 (10·7–13·9)	185 (6·9%)	9·9 (8·6–11·5)	25 (0·9%)	1·3 (0·9–2·0)
		>4·7 (most fragmented)	1719 (9·4%)	143 (8·3%)	12·1 (10·3–14·3)	112 (6·5%)	9·5 (7·9–11·4)	19 (1·1%)	1·6 (1·0–2·5)
	Ethnic density (% ethnic minorities)								
		0·3 (lowest ethnic density)	1665 (9·1%)	138 (8·3%)	16·6 (14·0–19·7)	107 (6·4%)	12·9 (10·7–15·6)	23 (1·4%)	2·8 (1·8–4·2)
		19·0	1783 (9·8%)	189 (10·6%)	16·3 (14·1–18·8)	154 (8·6%)	13·3 (11·3–15·5)	21 (1·2%)	1·8 (1·2–2·8)
		28·7	1788 (9·8%)	187 (10·5%)	15·2 (13·2–17·6)	152 (8·5%)	12·4 (10·6–14·5)	20 (1·1%)	1·6 (1·1–2·5)
		35·0	1765 (9·7%)	173 (9·8%)	13·8 (11·8–16·0)	135 (7·6%)	10·7 (9·1–12·7)	19 (1·1%)	1·5 (1·0–2·4)
		39·6	1861 (10·2%)	171 (9·2%)	13·2 (11·4–15·4)	135 (7·3%)	10·5 (8·8–12·4)	24 (1·3%)	1·9 (1·2–2·8)
		43·8	1851 (10·2%)	208 (11·2%)	16·0 (14·0–18·3)	166 (9·0%)	12·8 (11·0–14·9)	22 (1·2%)	1·7 (1·1–2·6)
		48·4	1840 (10·1%)	190 (10·3%)	14·6 (12·7–16·8)	149 (8·1%)	11·5 (9·8–13·5)	15 (0·8%)	1·2 (0·7–1·9)
		53·3	1884 (10·4%)	181 (9·6%)	13·5 (11·7–15·6)	146 (7·7%)	10·9 (9·3–12·8)	18 (1·0%)	1·3 (0·8–2·1)
		60·0	1872 (10·3%)	162 (8·7%)	12·5 (10·8–14·6)	137 (7·3%)	10·6 (9·0–12·5)	11 (0·6%)	0·9 (0·5–1·5)
		67·1 (highest ethnic density)	1892 (10·4%)	168 (8·9%)	13·2 (11·3–15·4)	136 (7·2%)	10·7 (9·0–12·6)	19 (1·0%)	1·5 (1·0–2·3)
	Individual-level indicators								
	Age at diagnosis (years)*								
		<38·1	8723 (47·9%)	230 (2·6%)	3·92 (3·4–4·5)	121 (1·4%)	2·1 (1·7–2·5)	92 (1·1%)	1·6 (1·3–1·9)
		≥38·2	9478 (52·1%)	1537 (16·2%)	24·0 (22·8–25·2)	1296 (13·7%)	20·2 (19·2–21·4)	100 (1·1%)	1·6 (1·3–1·9)
	Gender								
		Males	9610 (52·8%)	908 (9·4%)	13·4 (12·6–14·3)	707 (7·4%)	10·4 (9·7–11·2)	132 (1·4%)	1·9 (1·6–2·3)
		Females	8591 (47·2%)	859 (10·0%)	15·6 (14·6–16·7)	710 (8·3%)	12·9 (12·0–13·9)	60 (0·7%)	1·1 (0·8–1·4)
	Diagnosis								
		Non-affective	13 160 (72·3%)	1358 (10·3%)	14·7 (14·0–15·5)	1090 (8·3%)	11·8 (11·1–12·5)	141 (1·1%)	1·5 (1·3–1·8)
		Affective	5041 (27·7%)	409 (8·1%)	13·4 (12·2–14·8)	327 (6·5%)	10·7 (9·6–11·9)	51 (1·0%)	1·7 (1·3–2·2)
	Marital status								
		Married or cohabiting	2781 (15·3%)	267 (9·6%)	15·8 (14·0–17·8)	220 (7·9%)	13·0 (11·4–14·9)	22 (0·8%)	1·3 (0·9–2·0)
		Divorced, separated, widowed, or single	15 420 (84·7%)	1500 (9·7%)	14·2 (13·5–14·9)	1197 (7·8%)	11·3 (10·7–12·0)	170 (1·1%)	1·6 (1·4–1·9)
	Substance use disorder								
		No substance use disorder	15 046 (82·7%)	1519 (10·1%)	15·1 (14·3–15·8)	1251 (8·3%)	12·4 (11·7–13·1)	128 (0·9%)	1·3 (1·1–1·5)
		Life-time substance use disorder	3155 (17·3%)	248 (7·9%)	11·3 (10·0–12·9)	166 (5·3%)	7·6 (6·5–8·8)	64 (2·0%)	2·9 (2·3–3·7)
	Ethnicity								
		White British	9047 (49·7%)	1130 (12·5%)	19·9 (18·8–21·1)	913 (10·1%)	16·1 (15·1–17·2)	125 (1·4%)	2·2 (1·8–2·6)
		Ethnic minorities	9154 (50·3%)	637 (7·0%)	9·7 (8·9–10·4)	504 (5·5%)	7·6 (7·0–8·3)	67 (0·7%)	1·0 (0·8–1·3)

Data are n (%), unless otherwise specified. Crude rates are per 1000 person-years. 158 deaths were from causes not otherwise classified (R00-R99) and contribute to all-cause mortality totals in the sample.

* Age was handled as a time-varying covariate in all regression models and is displayed here divided at the median.

Considerable variation was observed across the sample for area-level indicators. For example, urbanicity ranged from 36·7 persons per hectare (10th percentile) to 173·1 persons per hectare (90th percentile). Ethnic density ranged from 20·3% ethnic minorities (10th percentile) to 67·1% ethnic minorities (90th percentile). Both variables were normally distributed.

When compared with the total population of England and Wales, the age-standardised and gender-standardised mortality ratios were higher for the sample irrespective of the neighbourhood-level characteristics of areas of residence (appendix). Standardised mortality ratios by each area-level characteristic were broadly similar with overlapping 95% CIs. Deaths from all causes and natural causes were elevated approximately 2–3 times in the sample, whereas deaths from unnatural causes were elevated around 4–7 times in the sample, when age-standardised and gender-standardised to the population of England and Wales.

In the cohort with severe mental illness, we found that none of the area-level indicators for deprivation, urbanicity, or social fragmentation appeared to have an association with any of the mortality risks, across adjusted models (Table 2, Table 3, Table 4), although there were noteworthy associations for individual-level variables with causes of death. Women with severe mental illness had a lower risk of death from all causes and from unnatural causes than in men, the presence of an affective diagnosis compared with non-affective was associated with a lower risk of death from all causes and from natural causes, and people with severe mental illness who were divorced or in disrupted relationships had a higher risk of death from all causes and from natural causes than in those with severe mental illness who were married or cohabiting. A history of current or previous substance use disorders was associated with an increased risk of death from all causes and from unnatural causes in the cohort with severe mental illness (Table 2, Table 3, Table 4).

**Table 2:** Association of area-level and individual-level indicators with all-cause mortality in people with severe mental illness

		**Total sample (n=18 201)**	**All-cause deaths (n=1767)**	**Model 1**		**Model 2**	
				Incidence risk ratios (95% CI)	p value	Adjusted incidence risk ratios (95% CI)	p value
**Area level indicators**							
Index of Multiple Deprivation (per increase in fifths; from less to more deprived)		..	..	0·98 (0·95–1·02)	0·34*	1·03 (0·99–1·07)	0·19*
Urbanicity (per increase in fifths; from less to more urban)		..	..	0·95 (0·92–0·99)	0·01*	0·97 (0·93–1·01)	0·14*
Social fragmentation (per unit increase; from less to more fragmented)		..	..	0·95 (0·92–1·00)	0·03*	0·98 (0·94–1·03)	0·45*
**Individual-level indicators**							
Gender							
	Male	9610 (52·8%)	908 (51·4%)	Ref		Ref	
	Female	8591 (47·2%)	859 (48·6%)	1·07 (0·98–1·18)	0·13	0·86 (0·78–0·94)	0·0015
Diagnosis							
	Non-affective	13 160 (72·3%)	1358 (76·9%)	Ref		Ref	
	Affective	5041 (27·7%)	409 (23·1%)	0·82 (0·73–0·91)	<0·0001	0·83 (0·74–0·93)	0·0015
Marital status							
	Married or cohabiting	2781 (15·3%)	267 (15·1%)	Ref		Ref	
	Divorced, separated, widowed, or single	15 420 (84·7%)	1500 (84·9%)	1·01 (0·89–1·15)	0·89	1·28 (1·12–1·46)	<0·0001
Substance use disorder							
	No substance disorder	15 046 (82·7%)	1519 (86·0%)	Ref		Ref	
	Life-time substance use disorder	3155 (17·3%)	248 (14·0%)	0·79 (0·69–0·90)	<0·001	1·17 (1·02–1·35)	0·024
**Interaction of ethnicity with areal ethnic density**							
Lowest ethnic density area (0% ethnic minorities)							
	White British	..	..	Ref		Ref	
	Ethnic minorities	..	..	0·88 (0·65–1·17)	0·38	0·96 (0·71–1·29)	0·77
Highest ethnic density area (95% ethnic minorities)							
	White British	..	..	Ref		Ref	
	Ethnic minorities	..	..	0·31 (0·23–0·43)	<0·0001	0·52 (0·38–0·71)	<0·0001
p value between ethnicity and ethnic density interaction		..	..	..	<0·0001	..	0·036

Data are n (%), unless otherwise specified. Model 1 is crude estimates. Model 2 is adjusted for age, interaction between area-level ethnic density and ethnicity, and all other displayed variables. p values are from Wald tests.

* p value for linear trend.

**Table 3:** Sub-hazard ratio estimates for natural-cause mortality in people with severe mental illness

		**Total sample (n=18 201)**	**Natural-cause deaths (n=1417)**	**Model 1**		**Model 2**	
				Sub-hazard ratios (95% CI)	p value	Sub-hazard ratios (95% CI)	p value
**Area-level indicator**s							
Index of Multiple Deprivation (per increase in fifths; from less to more deprived)		..	..	0·95 (0·91–1·00)	0·05*	1·02 (0·97–1·06)	0·49*
Urbanicity (per increase in fifths; from less to more urban)		..	..	0·94 (0·90–0·98)	0·005*	0·99 (0·94–1·03)	0·55*
Social fragmentation (per unit increase; from less to more fragmented)		..	..	0·93 (0·88–0·97)	0·002*	0·98 (0·93–1·03)	0·41*
**Individual-level indicators**							
Gender							
	Male	9610 (52·8%)	707 (49·9%)	Ref		Ref	
	Female	8591 (47·2%)	710 (50·1%)	1·25 (1·11–1·39)	<0·0001	0·92 (0·82–1·02)	0·11
Diagnosis							
	Non-affective	13 160 (72·3%)	1090 (76·9%)	Ref		Ref	
	Affective	5041 (27·7%)	327 (23·1%)	0·90 (0·80–1·02)	0·11	0·82 (0·73–0·93)	0·002
Marital status							
	Married or cohabiting	2781 (15·3%)	220 (15·5%)	Ref		Ref	
	Divorced, separated, widowed, or single	15 420 (84·7%)	1197 (84·5%)	0·87 (0·74–1·01)	0·07	1·16 (1·00–1·35)	0·05
Substance use disorder							
	No substance use disorder	15 046 (82·7%)	1251 (88·3%)	Ref		Ref	
	Life-time substance use disorder	3155 (17·3%)	166 (11·7%)	0·61 (0·52–0·71)	<0·0001	0·91 (0·77–1·06)	0·22
**Interaction of ethnicity with areal ethnic density**							
Lowest ethnic density area (0% ethnic minorities)							
	White British	..	..	Ref		Ref	
	Ethnic minorities	..	..	0·69 (0·51–0·94)	0·019	0·78 (0·57–1·05)	0·11
	Highest ethnic density area (95% ethnic minorities)						
	White British	..	..	Ref		Ref	
	Ethnic minorities	..	..	0·31 (0·22–0·44)	<0·0001	0·44 (0·32–0·62)	<0·0001
p value between ethnicity and ethnic density interaction		..	..	..	0·013	..	0·071

Data are n (%), unless otherwise specified. Competing risks regression models with robust standard errors to adjust for clustering at Lower Super Output Area level were used to estimate sub-hazard ratios. Model 1 is crude estimates. Model 2 is adjusted for age, an interaction between area level own ethnic density and ethnicity, and all other displayed variables. p values are from Wald tests.

* p value for linear trend.

**Table 4:** Sub-hazard ratio estimates for unnatural-cause mortality in people with severe mental illness

		**Total sample (n=18 201)**	**Unnatural-cause deaths (n=192)**	**Model 1**		**Model 2**	
				Sub-hazard ratios (95% CI)	p value	Sub-hazard ratios (95% CI)	p value
**Area level indicators**							
Index of Multiple Deprivation (per increase in fifths; from less to more deprived)		..	..	0·88 (0·80–0·98)	0·02*	0·94 (0·83–1·06)	0·32*
Urbanicity (per increase in fifths; from less to more urban)		..	..	0·87 (0·79–0·97)	0·01*	0·94 (0·83–1·07)	0·33*
Social fragmentation (per unit increase; from less to more fragmented)		..	..	0·98 (0·87–1·10)	0·70*	1·02 (0·90–1·15)	0·80*
**Individual-level indicators**							
Gender							
	Male	9610 (52·8%)	132 (68·8%)	Ref		Ref	
	Female	8591 (47·2%)	60 (31·3%)	0·54 (0·40–0·73)	<0·0001	0·61 (0·44–0·84)	0·0026
Diagnosis							
	Non-affective	13 160 (72·3%)	141 (73·4%)	Ref		Ref	
	Affective	5041 (27·7%)	51 (26·6%)	1·05 (0·76–1·45)	0·75	1·01 (0·73–1·41)	0·95
Marital status							
	Married or cohabiting	2781 (15·3%)	22 (11·5%)	Ref		Ref	
	Divorced, separated, widowed, or single	15 420 (84·7%)	170 (88·5%)	1·29 (0·83–2·00)	0·26	1·08 (0·69–1·68)	0·75
Substance use disorder							
	No substance use disorder	15 046 (82·7%)	128 (66·7%)	Ref		Ref	
	Life-time substance use disorder	3155 (17·3%)	64 (33·3%)	2·36 (1·74–3·20)	<0·0001	2·07 (1·52–2·81)	<0·0001
**Interaction of ethnicity with areal ethnic density**							
Lowest ethnic density area (0% ethnic minorities)							
	White British	..	..	Ref		Ref	
	Ethnic minorities	..	..	0·57 (0·24–1·39)	0·22	0·52 (0·21–1·26)	0·15
	Highest ethnic density area (95% ethnic minorities)						
	White British	..	..	Ref		Ref	
	Ethnic minorities	..	..	0·47 (0·17–1·30)	0·15	0·51 (0·19–1·40)	0·19
p value between ethnicity and ethnic density interaction		..	..	..	0·84	..	0·99

Data are n (%), unless otherwise specified. Competing risks regression models with robust standard errors to adjust for clustering at Lower Super Output Area level were used to estimate sub-hazard ratios. Model 1 is crude estimates. Model 2 is adjusted for age, an interaction between area level own ethnic density and ethnicity, and all other displayed variables. p values are from Wald tests.

* p value for linear trend.

We assessed the cross-level interaction of individual-level ethnicity (all of the ethnic minority groups in the study aggregated) with area-level total ethnic density across mortality outcomes. Significant ethnicity to ethnic density cross-level interactions were noted for all-cause mortality (figure). In the least ethnically dense areas (lowest decile category range of 0·30–19% ethnic minorities, adjusted incidence risk ratios [as] estimated at 0% ethnic minorities), individuals with severe mental illness, belonging to an ethnic minority group, had a similar adjusted rate ratio (aRR) of death compared with white British individuals with severe mental illness (aRR 0·96 95% CI 0·71–1·29); whereas in the highest ethnic density areas (highest decile category range of 67·196·4%, aIRR estimated at 95% ethnic minorities) the relative risk reduced to half that of white British individuals with severe mental illness (aRR 0·52, 95% CI 0·38–0·71), with strong evidence in support of a statistical interaction between individual-level ethnicity and area-level ethnic density (p=0·036 for statistical interaction; table 2; figure). A similar trend was noted for deaths from natural causes although with weaker evidence in support of a cross-level statistical interaction (p=0·071 for statistical interaction; table 3; figure). No cross-level interactions were noted for deaths from unnatural causes (table 4; figure).

**Figure:**
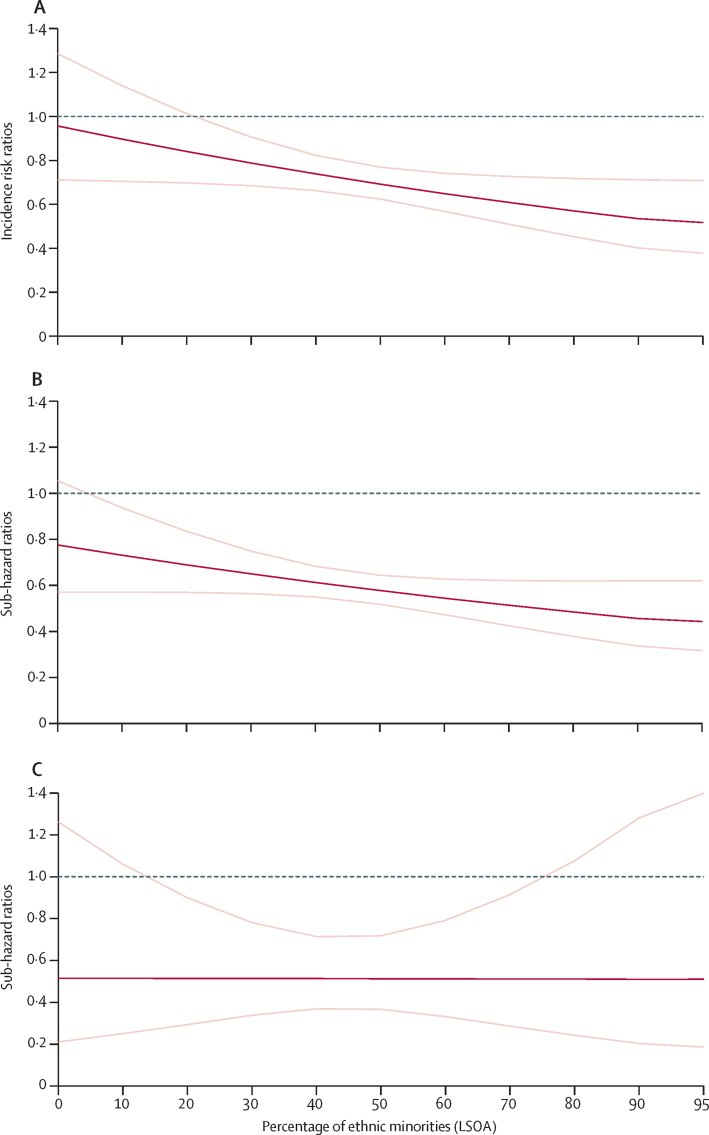
Ethnic density associations at LSOA level (n=18 201) (A) Adjusted incidence risk ratios for all-cause mortality by ethnic density. (B) Adjusted sub-hazard ratios for natural-cause mortality by ethnic density. (C) Adjusted sub-hazard ratios for unnatural-cause mortality by ethnic density. Estimates are adjusted for area-level deprivation, urbanicity, social fragmentation, gender, diagnosis, marital status, substance use disorders, and age. p values for ethnicity × ethnic density interactions were p=0·036 for all-cause mortality, p=0·071 for natural-cause mortality, and p=0·99 for unnatural-cause mortality. Dark red lines show adjusted incidence risk ratios (all-cause mortality) or adjusted sub-hazard ratios (natural-cause or unnatural-cause mortality) in ethnic minority groups relative to white British people with severe mental illness (reference group), with 95% CIs delineated by light red lines. Grey lines show incidence risk ratio or sub-hazard ratios of 1·00 for the reference group. LSOA=Lower Super Output Area.

We also assessed cross-level ethnicity and own-group ethnic density associations for each of the ethnic minority groups within the sample. In general, similar trends were supported across black African, black Caribbean, and south Asian groups with severe mental illness, but most pronounced in the south Asian group. In adjusted models, aRRs for deaths from all causes in south Asian people with severe mental illness were similar to white British people with severe mental illness in the lowest south Asian ethnic density areas (lowest decile category ranged from 0–4·1%, calculated at 0% south Asian ethnic density; aRR 1·08, 95% CI 0·76–1·54; p=0·67). This aRR reduced to 0·07 (95% CI 0·01–0·49; p=0·007) in the highest south Asian ethnic density areas (highest decile category ranged from 19·8% to 86·8%, calculated at 90% south Asian ethnic density). A similar trend was noted for deaths from natural causes for this group with severe mental illness (relative to white British people with severe mental illness, aRR was 1·03, 95% CI 0·73–1·45; p=0·88) in the areas of the lowest south Asian ethnic density, which had reduced to aRR 0·04 (95% CI 0·01–0·23; p<0·0001) in the areas of highest south Asian ethnic density. Similar, albeit weaker associations were noted for the black African group with severe mental illness, with similar trends for all-cause and natural-cause mortality in black Caribbean groups with severe mental illness (appendix).

Sensitivity analyses were run specifying the risk of emigration out of the cohort as a competing risk in regression models for all-cause mortality. On the whole, most associations across the cohort were retained. Ethnic density associations highlighting a reduced risk of all-cause mortality in areas of higher ethnic density in ethnic minority cohort members with severe mental illness (relative to white British people with severe mental illness) were still observed across models, although the strength of the evidence in support of the ethnicity and ethnic density cross-level interaction across the adjusted model was weaker (appendix).

## Discussion

We found that in areas of low ethnic density, risks for all-cause and natural-cause mortality were similar in ethnic minority groups with severe mental illness compared with white British people with severe mental illness. By contrast, in neighbourhoods of high ethnic density, risks for death from all causes and from natural causes were significantly reduced in ethnic minority groups with severe mental illness relative to white British people with severe mental illness. Similar trends were observed across most of the ethnic minority groups in the study, with the strongest associations noted for all-cause and natural-cause mortality in south Asian groups with severe mental illness compared with white British groups with severe mental illness. Residency in areas that were more deprived, urban, or socially fragmented did not appear to be associated with elevated all-cause, natural-cause, or unnatural-cause mortality in people with severe mental illness. People with severe mental illness in this study experienced a 2–3-times increased risk of all-cause and natural-cause mortality and 4–7-times increased risk of unnatural-cause mortality, when age-standardised and gender-standardised to a non-severe mental illness reference population. These differences persisted when compared across the different characteristics of the neighbourhoods surveyed.

Our literature reviews did not identify any studies that have previously directly assessed the association of residency in socially fragmented neighbourhoods with mortality in severe mental illness, although previous ecological studies have suggested an association between increasing social fragmentation and suicide mortality in the general population.^18^, ^20^ The lack of an association between social fragmentation and mortality in our study, in contrast to previous work, might be because previously noted associations were only observed at the whole-population level, whereas our study had a primary focus on a population with severe mental illness. Previous work might have also been affected by ecological bias, whereby inferences about individuals are erroneously determined from ecological or area-level characteristics. The differences between our study and this previous work might also reflect that we were able to include both individual-level and area-level covariates, avoiding such biases.

For area-level deprivation, research findings have been inconsistent. Although associations have been noted in some studies between residency in more deprived areas and increased mortality in severe mental illness,^3^, ^24^, ^25^ in one study from a UK nationally representative sample, the investigators reported that people with severe mental illness resident in more deprived areas had a lower risk of suicide than people resident in less deprived areas.^26^ Our findings might indicate that for ethnic minority groups with severe mental illness, area-level factors such as ethnic density, which might buffer against social isolation and be associated with enhanced social support^11^, ^12^ and social capital,^27^ might counteract any neighbourhood material advantages on health.

Markedly increased standardised mortality ratios observed in this cohort, when compared with the population without severe mental illness, are consistent with previous work in international samples, and underlie an urgent need to address premature mortality in people with severe mental illness.^1^, ^2^ Across international contexts, associations between residency in urban areas and mortality in people with severe mental illness have been mixed.^28^, ^29^ Investigators in the original WHO international studies of schizophrenia observed that all-cause mortality in people with severe mental illness was lower in less industrialised settings compared with industrialised settings,^4^ whereas investigators in a more recent systematic review did not find an association between urbanicity and mortality in severe mental illness.^30^ The notion that outcomes, including mortality, for people living with severe mental illness in developing versus developed countries are better has been critiqued.^8^ We did not find an association between urbanicity and mortality in severe mental illness in our study, although we observed that residency in areas of higher own-group ethnic density for some ethnic minority groups was associated with fewer adverse mortality outcomes in severe mental illness. An understanding of possible aetiological mechanisms underlying observed associations might be informed by previous research, in which the association of own ethnic density with physical health morbidity and mortality outcomes in populations without severe mental illness, has been explored.^31^ For example, in the UK, black African, black Caribbean, and south Asian individuals resident in areas of higher own-group density report lower levels of alcohol use.^32^ Mechanisms underlying these findings might include the mutual support of others of a similar ethnic background, as well as the health protective effects of social norms mitigating against adverse health-related behaviours.^31^ Conversely, residency in areas of low ethnic density for ethnic minority groups might be associated with lower social support, greater social isolation, and greater discrimination.^11^, ^12^ Therefore, the findings in the present study might not just be specific to severe mental illness but might reflect the wider effect of own-group ethnic density on health-related behaviours and mortality in general. The role of neighbourhood or group-level factors in accounting for mortality risks in severe mental illness could be an important area of future enquiry.^2^

Ethnic density trends were most notable for south Asian and black African groups. There is a large literature indicating poorer mental and physical health in Irish people in the UK,^33^ which for common mental disorders and suicidal ideation might be offset by residency in areas of higher own-group density;^12^, ^34^ however, this finding was not observed in the present study. Smaller sample sizes in the present study might have reduced the power to detect differences for Irish people with severe mental illness. In addition, unlike the other ethnic minority groups within the study, we found that whereas strong positive correlations were observed with own-group density and the total ethnic minority density variable for black African, black Caribbean, and south Asian groups, this finding was not observed for Irish own-group density (appendix), possibly reflecting differing historical patterns of settlement across each of these groups in the catchment area of the study. Within this catchment area, Irish groups with severe mental illness were resident in areas of lower total ethnic minority density and were more dispersed. This factor might be another reason why we did not find an ethnic density association for the Irish group and could be explored in larger nationally representative samples.

We used reliable methodologies to derive each of the area-level variables, which have been widely used previously, have a strong theoretical basis, and have been validated.^11^, ^16^, ^18^, ^19^ A strength of the present study was in the possibility of using both neighbourhood and individual-level variables. It may be a limitation that we assessed area-level factors at one timepoint only (time of diagnosis) and could not assess all individual-level variables at cohort entry. This may have led to random measurement error. If this was the case, then associations between exposures with outcomes may have been under-estimated. As we used address at the time of diagnosis, linked to area-level indicators from the study midpoint (2010–11), this may also have further contributed to imprecise estimates.

People with severe mental illness are more likely to experience downward social drift as their illness progresses. Such drift effects might also operate in the prodromal phase, before onset. A possible limitation of our methods is that the location of residency was obtained at the point of diagnosis. It is unclear how such drift effects might have biased our findings. As ethnically dense areas are also in general more deprived and urban, it is unlikely that individuals with severe mental illness from minority ethnic groups would be able to drift into ethnically less dense areas (as such areas would be expected to be more affluent and less urban). However, white British people with severe mental illness might drift into higher ethnic density areas. As we would also expect that ethnic minority groups with severe mental illness in low ethnic density areas could drift into higher ethnic density areas, it is difficult to quantify how far such drift effects would operate selectively for ethnic groups with severe mental illness, and how this effect would then affect the mortality findings.

Other limitations of the study included our inability to assess important individual-level variables such as health-related behaviours, physical health comorbidities, individual-level socioeconomic status, and social support. Work is underway to link the cohort to other data sources, which might eventually allow us directly to assess these indicators as potential confounders or mediators for mortality. A further limitation is that it was not possible to derive standardised mortality ratios by ethnicity, as this information is not routinely recorded in UK death certificates.^35^ Although the catchment area of the study is typical of many large urbanised locations in Britain and internationally, with potentially good generalisability to metropolitan locations elsewhere, it is a limitation that we were not able to include locations outside of the Greater London area, which could be considered in future work. We focused the analyses on key ethnic minority groups who were well represented within the catchment area of the study and have previously been included in national surveys of ethnic minority health; however, other ethnic minority groups were not included, such as those who might self-identify as “white other”. Future work could explore health inequalities affecting these groups. The southern part of the catchment area for this study included less urbanised areas and there is a possibility that overall there was inadequate variation in the degree of urbanisation to enable associations to be detected. However, previous work from this location has indicated “considerable heterogeneity” across this catchment area for ethnic density, population density, and other measures of social fragmentation such as voter turnout,^36^ which was also observed in the range of urbanicity and ethnic density variables in the present study. In addition, our scoping of the literature indicated inconsistent associations between residency in urban versus rural areas and mortality in severe mental illness,^28^, ^37^, ^38^ so our findings are not inconsistent with the broader literature; future work could explore these associations using national data. Finally, most of the ethnic density comparisons were made internally to the cohort with severe mental illness; therefore, it is possible that these findings reflect ethnic density associations for ethnic minority people in general and should be explored in the future using a population without severe mental illness.

For ethnic minority groups with severe mental illness, we might speculate that residency in areas of higher own-group density buffers against social exclusion, social isolation, and promotes health-protective behaviours, which might have a role in mitigating against premature mortality in severe mental illness. However, the study was unable to directly assess this hypothesis. Future research should explore the role of contextual factors in accounting for mortality outcomes in severe mental illness, which could be used to guide intervention development.^2^

## Data sharing

Data are owned by a third party South London and Maudsley Biomedical Research Centre Clinical Record Interactive Search tool that provides access to anonymised data derived from electronic medical records of the South London and Maudsley National Health Service Foundation Trust. These data can only be accessed by permitted individuals from within a secure firewall (ie, remote access is not possible, and the data cannot be sent elsewhere) in the same manner as the authors.
