# Supplementary material for: Ethnic density and other neighbourhood associations for mortality in severe mental illness: a retrospective cohort study with multi-level analysis from an urbanised and ethnically diverse location in the UK
Source: Lancet Psychiatry. 2019 Jun;6(6):506–17. doi: 10.1016/S2215-0366(19)30126-9 (PMC6551347; doi:10.1016/S2215-0366(19)30126-9)
Supplement: Supplementary appendix [file mmc1.pdf]

THE LANCET
Psychiatry

Supplementary appendix

This appendix formed part of the original submission and has been peer reviewed. We post it as supplied by the authors.

Supplement to: Das-Munshi J, Schofield P, Bhavsar V, et al. Ethnic density and other neighbourhood associations for mortality in severe mental illness: a retrospective cohort study with multi-level analysis from an urbanised and ethnically diverse location in the UK. *Lancet Psychiatry* 2019; published online May 13. http://dx.doi.org/10.1016/S2215-0366(19)30126-9.

Title: Ethnic density and other neighbourhood associations for mortality in severe mental illnesses: Retrospective cohort study with multi-level analysis from an urbanised and ethnically diverse location in the UK

Supplementary material

Figure s1: Flow chart of participants in the study

Figure s2: Association of ethnic density with all-cause, natural cause and unnatural cause mortality: Black African individuals with SMI/ Black African ethnic density

Figure s3: Association of ethnic density with all-cause, natural cause and unnatural cause mortality: Black Caribbean individuals with SMI/ Black Caribbean ethnic density

Figure s4: Association of ethnic density with all-cause, natural cause and unnatural cause mortality: South Asian individuals with SMI/ South Asian ethnic density

Figure s5: Association of ethnic density with all-cause, natural cause and unnatural cause mortality: Irish individuals with SMI/ Irish ethnic density

Table s1: Age and sex- standardised mortality ratios by area-level indicators

Table s2: Sub-Hazard Ratios (sHRs) for ethnicity/ ethnic density associations with all-cause mortality in people with severe mental illness

Table s3: Sub-Hazard Ratios (sHRs) for ethnicity/ ethnic density associations with natural cause mortality in people with severe mental illness

Table s4: Sub-Hazard Ratios (sHRs) for ethnicity/ ethnic density associations with unnatural cause mortality in people with severe mental illness

Table s5: Sub Hazard Ratios (sHRs) for all-cause mortality with emigrations out of the cohort specified as a competing risk (sensitivity analysis)

Figure s6: Scatter plots of own group density and total ethnic minority density

Table s6: Search strategies and results for 'research in context' section

Figure s1: Flow chart of participants into the study

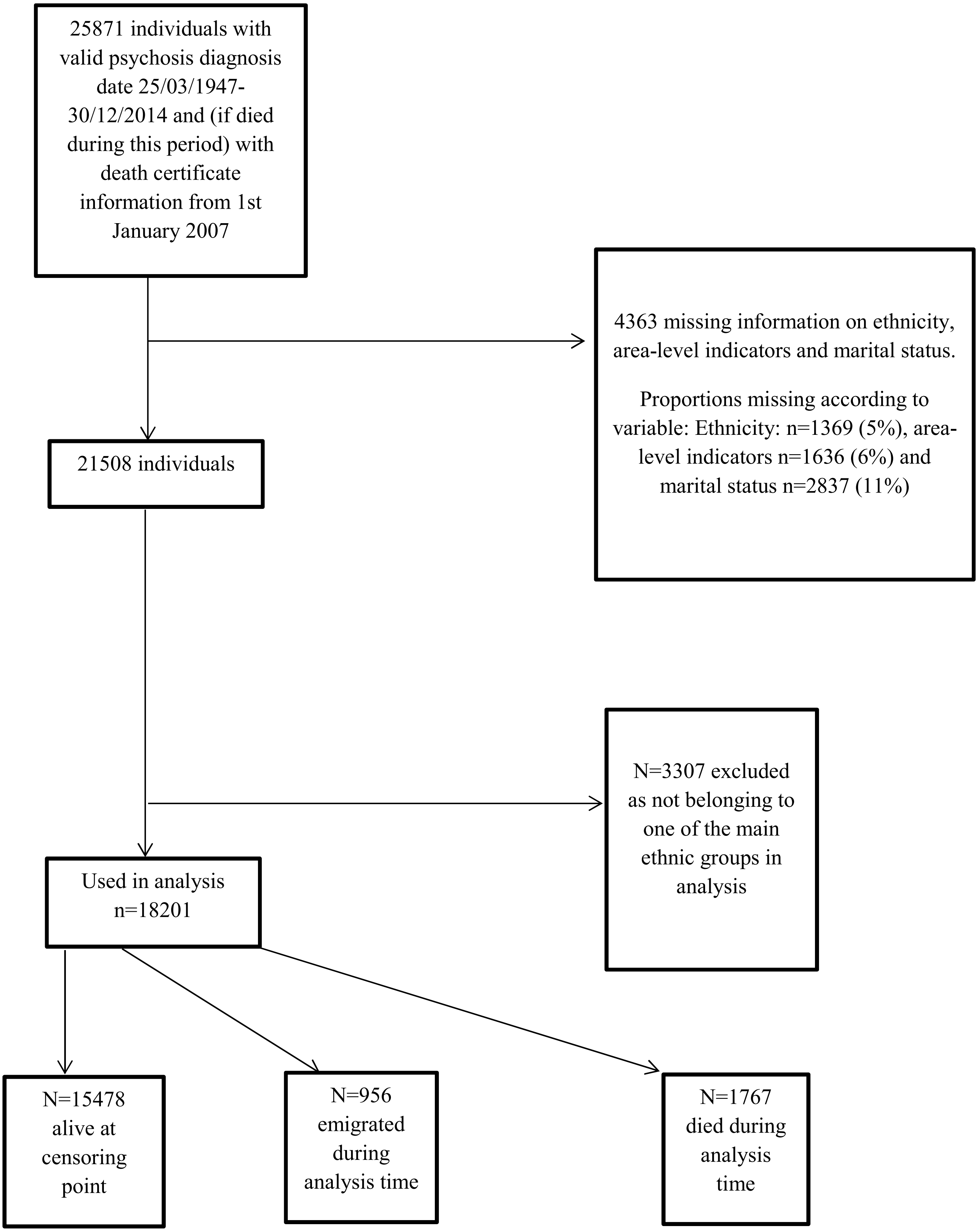

Figure S2: Interaction of Black African ethnicity with own-group ethnic density in SMI, for (1a) All-cause mortality; (1b) Natural cause mortality; (1c) Unnatural cause mortality

Figure S2a: Adjusted Rate Ratios (aRR) for all-cause mortality in SMI by own-group ethnic density (Black African)

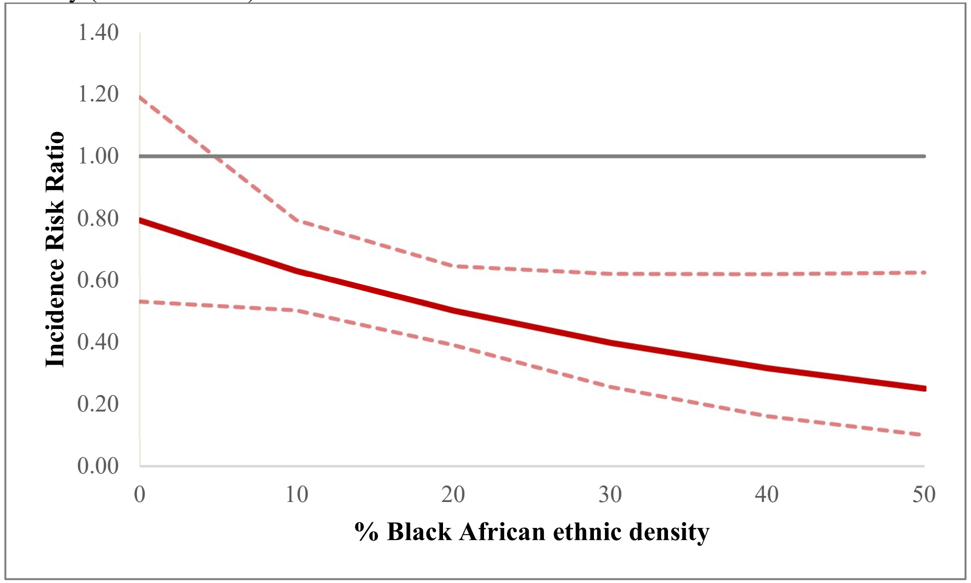

Figure S2b: Adjusted sub-Hazard Ratios (asHRs) for natural cause mortality in SMI by own-group density (Black African)

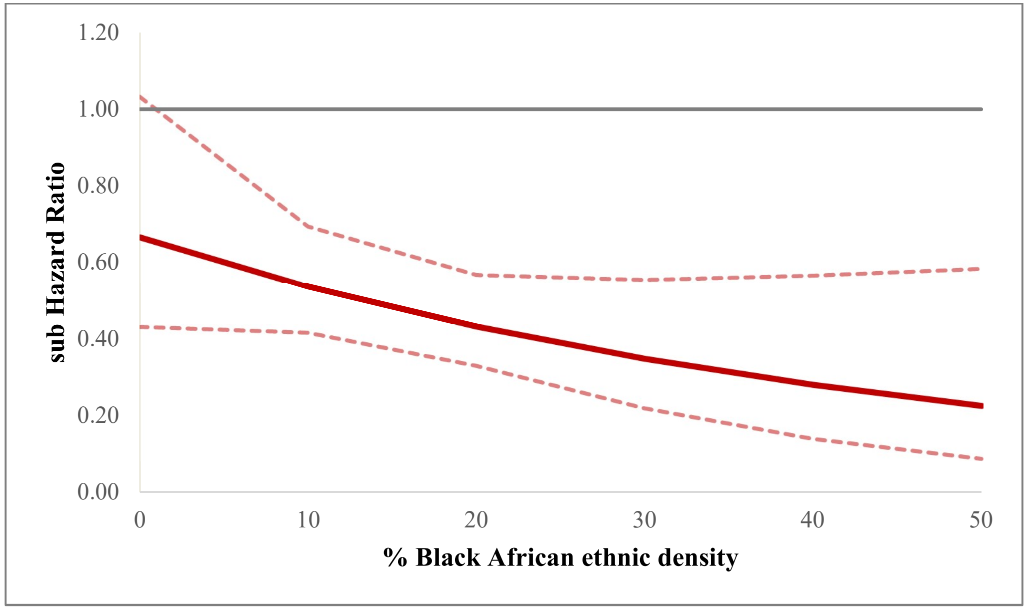

Figure S2c: Adjusted sub-Hazard Ratios (asHRs) for unnatural cause mortality in SMI by own-group density (Black African)

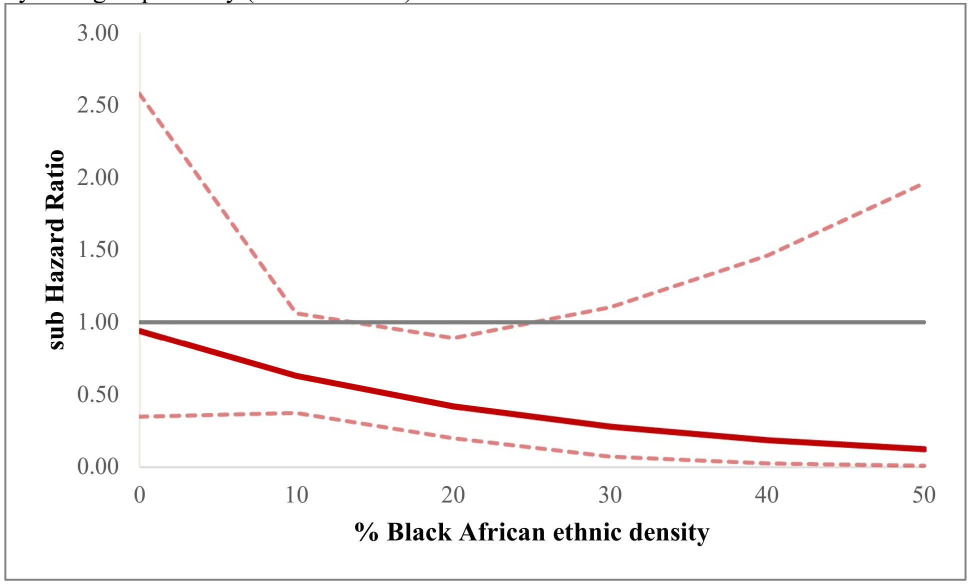

Legend

- (solid red line) aRR (All-cause mortality) or asHR (natural cause/ unnatural cause mortality) in Black African people with SMI
- (dashed line) 95% Confidence Intervals
- (grey line) Reference-White British group with SMI

Estimates are adjusted for area-level deprivation, urbanicity, social fragmentation, sex, diagnosis, marital status, substance use disorders and age.

P values for Black African ethnicity X Black African ethnic density interactions were: All-cause mortality: p=0.068; Natural cause mortality: p=0.10 and Unnatural cause mortality p=0.27

Total sample size 18201, with N=9047 White British and N=2510 Black African people

Figure S3: Interaction of Black Caribbean ethnicity with own-group ethnic density in SMI, for (1a) All-cause mortality; (1b) Natural cause mortality; (1c) Unnatural cause mortality

Figure S3a: Adjusted Rate Ratios (aRR) for all-cause mortality in SMI by own-group ethnic density (Black Caribbean)

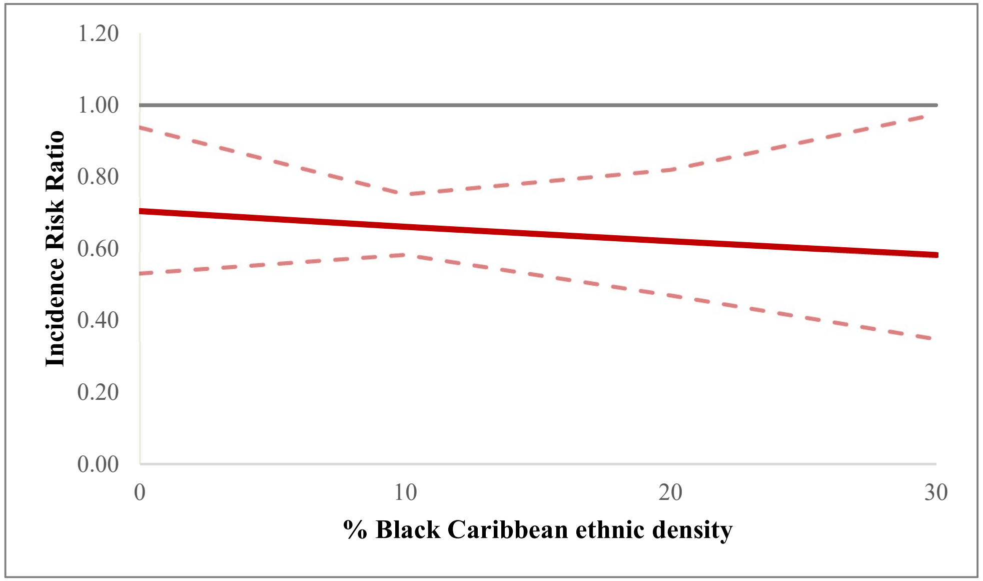

Figure S3b: Adjusted sub-Hazard Ratios (asHRs) for natural cause mortality in SMI by own-group density (Black Caribbean)

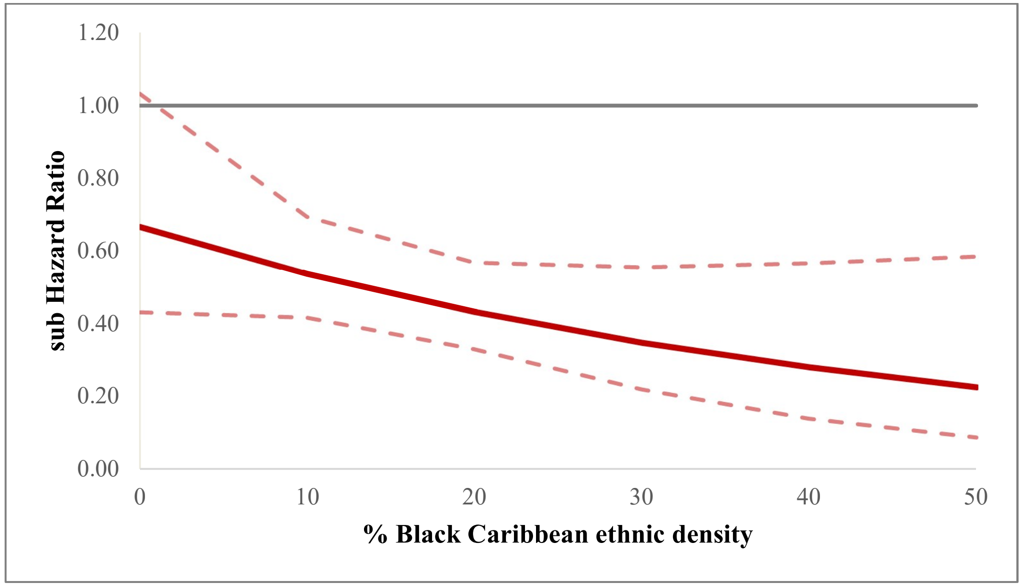

Figure S3c: Adjusted sub-Hazard Ratios (asHRs) for unnatural cause mortality in SMI by own-group density (Black Caribbean)

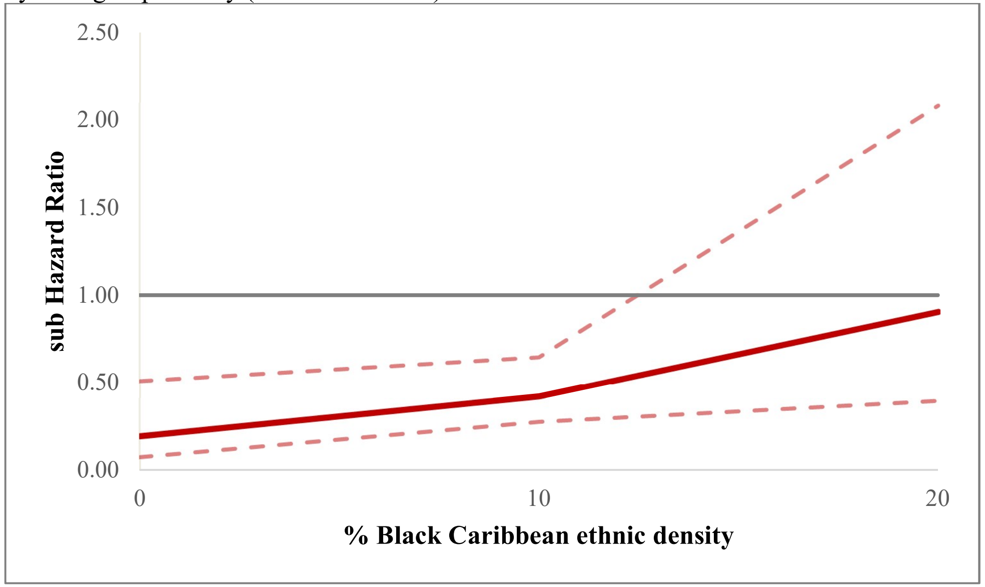

Legend

aRR (All-cause mortality) or asHR (natural cause/ unnatural cause mortality) in Black Caribbean people with SMI

95% Confidence Intervals

Reference-White British group with SMI

Estimates are adjusted for area-level deprivation, urbanicity, social fragmentation, sex, diagnosis, marital status, substance use disorders and age.

P values for Black Caribbean ethnicity X Black Caribbean ethnic density interactions were: All-cause mortality: p=0.62; Natural cause mortality: p=0.61 and Unnatural cause mortality p=0.056

Total sample size 18201, with N=9047 White British and N=4840 Black Caribbean people

Figure S4: Interaction of South Asian ethnicity with own-group ethnic density in SMI, for (1a) All-cause mortality; (1b) Natural cause mortality; (1c) Unnatural cause mortality

Figure S4a: Adjusted Rate Ratios (aRR) for all-cause mortality in SMI by own-group ethnic density (South Asian)

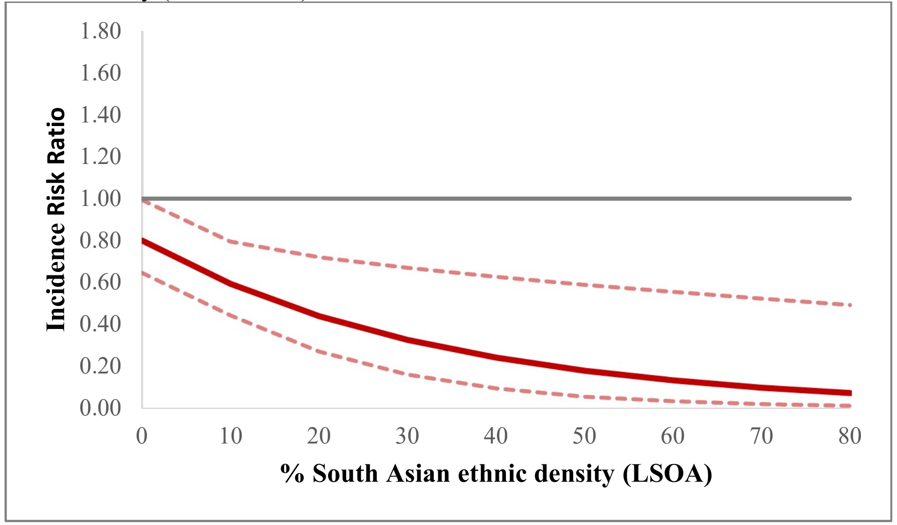

Figure S4b: Adjusted sub-Hazard Ratios (asHRs) for natural cause mortality in SMI by own-group density (South Asian)

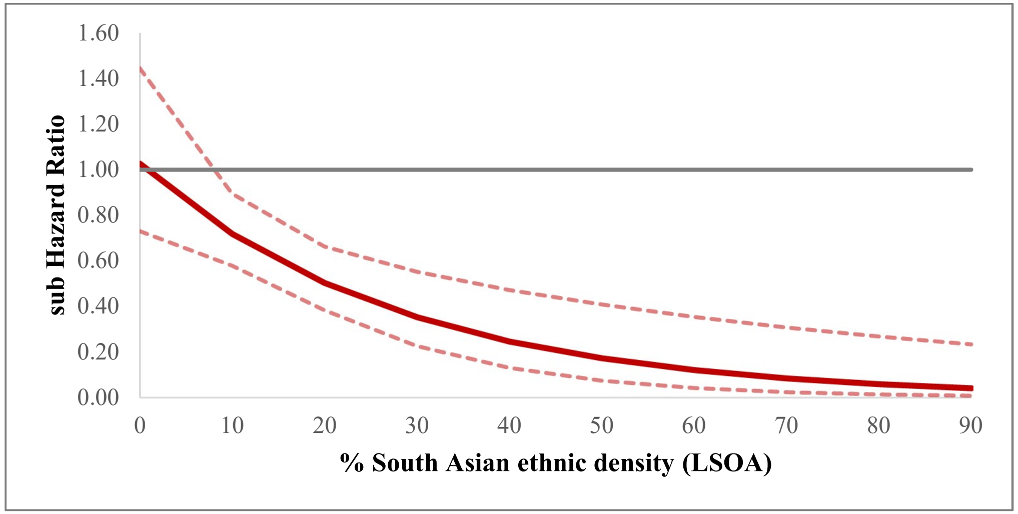

Figure S4c: Adjusted sub-Hazard Ratios (asHRs) for unnatural cause mortality in SMI by own-group density (South Asian)

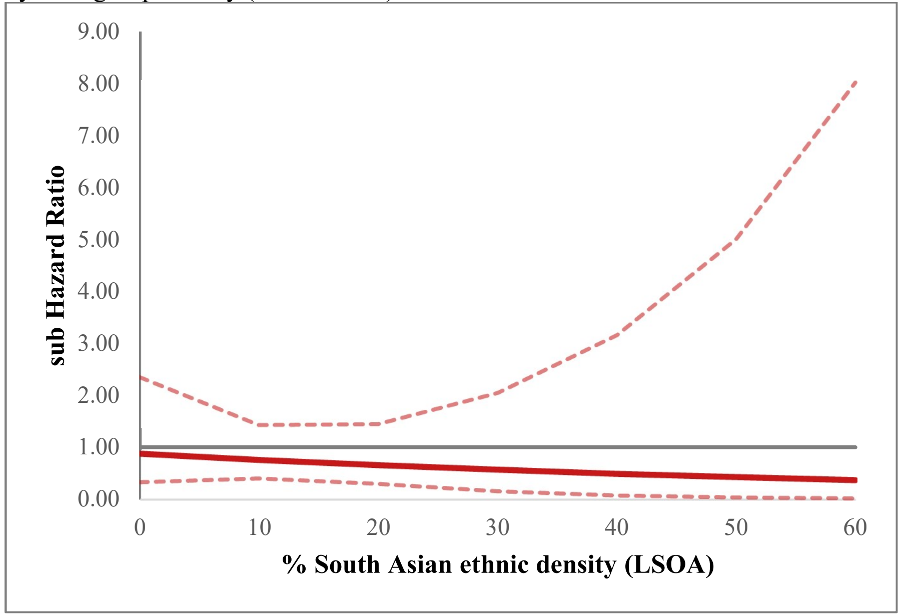

Legend

aRR (All-cause mortality) or asHR (natural cause/ unnatural cause mortality) in South Asian people with SMI

95% Confidence Intervals

Reference-White British group with SMI

Estimates are adjusted for area-level deprivation, urbanicity, social fragmentation, sex, diagnosis, marital status, substance use disorders and age.

P values for South Asian ethnicity X South Asian ethnic density interactions were: All-cause mortality: p=0.015; Natural cause mortality: p=0.001 and Unnatural cause mortality p=0.65

Total sample size 18201, with N=9047 White British and N=1256 South Asian people

Figure S5: Interaction of Irish ethnicity with own-group ethnic density in SMI, for (1a) All-cause mortality; (1b) Natural cause mortality; (1c) Unnatural cause mortality

Figure S5a: Adjusted Rate Ratios (aRR) for all-cause mortality in SMI by own-group ethnic density (Irish)

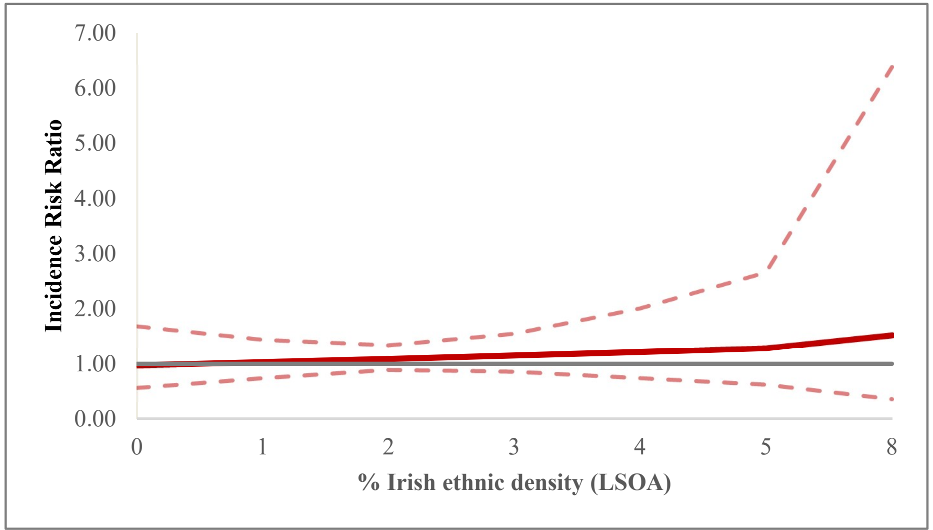

Figure S5b: Adjusted sub-Hazard Ratios (asHRs) for natural cause mortality in SMI by own-group density (Irish)

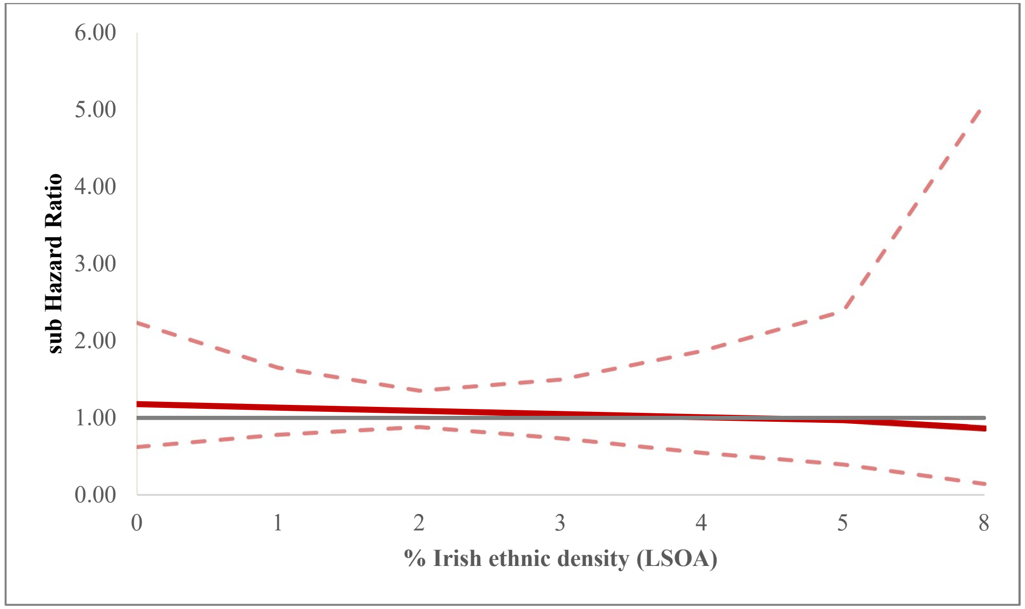

Figure S5c: Adjusted sub-Hazard Ratios (asHRs) for unnatural cause mortality in SMI by own-group density (Irish)

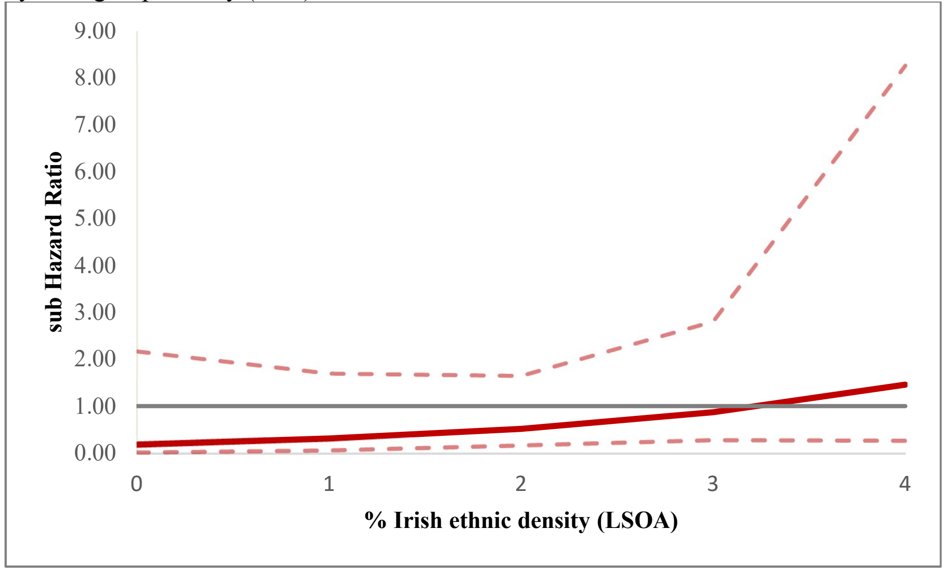

Legend

aRR (All-cause mortality) or asHR (natural cause/ unnatural cause mortality) in Irish people with SMI

95% Confidence Intervals

Reference-White British group with SMI

Estimates are adjusted for area-level deprivation, urbanicity, social fragmentation, sex, diagnosis, marital status, substance use disorders and age.

P values for Irish ethnicity X Irish ethnic density interactions were: All-cause mortality: p=0.65; Natural cause mortality: p=0.80 and Unnatural cause mortality p=0.26

Total sample size 18201, with N=9047 White British and N=548 Irish people

Supplementary Table S1: Age and sex- standardised mortality ratios by area-level indicators

	All-cause mortality		Natural cause mortality		Unnatural cause mortality	
	Observed/ Expected deaths	***SMR (95% CI)***	***Observed/ Expected deaths***	***SMR (95% CI)***	***Observed/ Expected deaths***	***SMR (95% CI)***
**Index of Multiple Deprivation**						
Least deprived	364/146	2.49 (2.24, 2.76)	295/ 141	2.10 (1.86, 2.35)	48/6	7.41 (5.46, 9.83)
Most deprived	377/132	2.86 (2.58, 3.16)	305/ 138	2.22 (1.97, 2.48)	33/7	4.47 (3.07, 6.27)
**Urbanicity**						
Lowest quintile	377/140	2.70 (2.43, 2.98)	301/137	2.20 (1.96, 2.46)	42/7	6.24 (4.50, 8.43)
Highest quintile	399/122	2.45 (2.18, 2.75)	244/126	1.93 (1.70, 2.19)	31/7	4.46 (3.03, 6.33)
**Social fragmentation**						
Lowest quintile						
Highest quintile	451/188	2.40 (2.18, 2.63)	364/181	2.00 (1.80, 2.22)	51/8	5.92 (4.41, 7.78)
	143/52	2.77 (2.34, 3.26)	112/55	2.03 (1.68, 2.45)	19/3	5.78 (3.48, 9.03)
**Ethnic density***						
Lowest density	152/52	2.90 (2.46, 3.40)	116/52	2.25 (1.86, 2.07)	24/3	8.44 (5.40, 12.55)
Highest density	169/55	3.06 (2.61, 3.55)	136/58	2.35 (1.97, 2.78)	19/3	5.50 (3.31, 8.60)

Key: **Lowest ethnic density areas ranged from 0.3 to 20.0%, highest ethnic density areas ranged from 67.1 to 96.4% ethnic minority groups*

Supplementary Table S2: Incidence Risk Ratios (IRR) for ethnicity/ ethnic density associations with all-cause mortality in people with severe mental illnesses

						Model 1			Model 2		
	N	Deaths	Person years (per 1000)	Crude rate (95% CI)		RR	95% CI	p value	RR	95% CI	p value
White British	9047	1130	56.79	19.9 (18.8, 21.1)	White British	REF			REF		
Black African	2510	106	16.41	6.5 (5.3, 7.8)	Black African, 0% own ethnic density	0.82	0.55 ,1.23	0.34	0.79	0.53 ,1.19	0.27
					Black African, 50% own ethnic density	0.23	0.09, 0.57	0.002	0.25	0.10, 0.62	0.003
					p-value for interaction			*0.043*			*0.068*
White British	9047	1130	56.79	19.9 (18.8, 21.1)	White British	REF			REF		
Black Caribbean	4840	332	37.71	8.8 (7.9, 9.8)	Black Caribbean, 0% own ethnic density	0.75	0.57 ,1.00	0.049	0.70	0.53 ,0.94	0.016
					Black Caribbean 30% own ethnic density	0.57	0.34, 0.95	0.03	0.58	0.35, 0.97	0.040
					p-value for interaction			*0.47*			*0.62*
White British	9047	1130	56.79	19.9 (18.8, 21.1)	White British	REF			REF		
South Asian	1256	95	8.06	11.8 (9.6, 14.4)	South Asian, 0% own ethnic density	1.06	0.75 ,1.50	0.75	1.08	0.76, 1.54	0.67
					South Asian, 90% own ethnic density	0.07	0.01, 0.44	0.005	0.07	0.01, 0.49	0.007
					p-value for interaction			*0.012*			*0.015*
White British	9047	1130	56.79	19.9 (18.8, 21.1)	White British	REF			REF		
Irish	548	104	3.8	27.7 (22.8, 33.5)	Irish, 0% own ethnic density	1.01	0.58 ,1.74	0.98	0.97	0.56, 1.68	0.93
					Irish, 11% own ethnic density	1.74	0.20, 14.95	0.62	1.80	0.21, 15.45	0.59
					p-value for interaction			*0.69*			*0.65*

*Model 1: Adjusted for age and sex, with an interaction for ethnicity*own ethnic density; Model 2: Adjusted for age, sex, area-level deprivation, urbanicity, social fragmentation, diagnosis, marital status and substance use disorders and ethnicity*own ethnic density interaction; P values from Wald tests*

Supplementary Table S3: Sub-Hazard Ratios (sHR) for ethnicity/ ethnic density associations with natural cause mortality in people with severe mental illness

						Model 1			Model 2		
	**N**	**Deaths**	**Person years (per 1000)**	**Crude rate (95% CI)**		**sHR**	**95% CI**	**p value**	**sHR**	**95% CI**	**p value**
White British	9047	913	4.23	215.8 (202.3, 230.3)	White British	REF			REF		
Black African	2510	82	0.33	250.50 (201.8 311.0)	Black African, 0% own ethnic density	0.70	0.46 , 1.07	0.099	0.67	0.43 , 1.03	0.069
					Black African, 50% own ethnic density	0.23	0.09 , 0.58	0.002	0.23	0.09 , 0.58	0.002
					p-value for interaction			*0.08*			*0.10*
White British	9047	913	4.23	215.8 (202.3, 230.3)	White British	REF			REF		
Black Caribbean	4840	256	1.37	186.4 (164.9, 210.7)	Black Caribbean, 0% own ethnic density	0.59	0.44 , 0.79	<0.0001	0.55	0.41 , 0.74	<0.0001
					Black Caribbean 30% own ethnic density	0.46	0.27 , 0.77	0.003	0.45	0.27 , 0.76	0.003
					p-value for interaction			*0.54*			*0.61*
White British	9047	913	4.23	215.8 (202.3, 230.3)	White British	REF			REF		
South Asian	1256	77	0.37	207.4 (165.9, 259.3)	South Asian, 0% own ethnic density	1.04	0.74 , 1.47	0.81	1.03	0.73 , 1.45	0.88
					South Asian, 90% own ethnic density	0.04	0.01 , 0.22	<0.0001	0.04	0.01 , 0.23	<0.0001
					p-value for interaction			*0.001*			*0.001*
White British	9047	913	4.23	215.8 (202.3, 230.3)	White British	REF			REF		
Irish	548	89	0.42	213.5 (173.4, 262.8)	Irish, 0% own ethnic density	1.19	0.63 , 2.24	0.59	1.18	0.62 , 2.23	0.61
					Irish, 11% own ethnic density	0.71	0.05 , 9,91	0.80	0.77	0.05 , 10.88	0.85
					p-value for interaction			*0.75*			*0.80*

*Model 1: Adjusted for age and sex, with an interaction for ethnicity*own ethnic density; Model 2: Adjusted for age, sex, area-level deprivation, urbanicity, social fragmentation, diagnosis, marital status and substance use disorders and ethnicity*own ethnic density interaction; P values from Wald tests*

Supplementary Table S4: sub-Hazard Ratios (sHRs) for ethnicity/ ethnic density associations with unnatural cause mortality in people with severe mental illness

						Model 1				Model 2			
	N	Deaths	Person years (per 1000)	Crude rate (95% CI)		sHR	95% CI		p value	sHR	95% CI		p value
White British	9047	125	0.44	284.7 (239.0, 339.3)	White British	REF				REF			
Black African	2510	18	0.06	306.0 (192.8, 485.7)	Black African, 0% own ethnic density	0.83	0.30	, 2.31	0.73	0.94	0.34	, 2.58	0.91
					Black African, 50% own ethnic density	0.10	0.01	, 1.75	0.12	0.12	0.01	, 1.96	0.14
					p-value for interaction				*0.26*				*0.27*
White British	9047	125	0.44	284.7 (239.0, 339.3)	White British	REF				REF			
Black Caribbean	4840	33	0.16	207.7 (147.7, 292.2)	Black Caribbean, 0% own ethnic density	0.18	0.07	, 0.47	<0.0001	0.20	0.08	, 0.51	0.001
					Black Caribbean 30% own ethnic density	1.94	0.39	, 9.68	0.42	1.95	0.41	, 9.29	0.40
					p-value for interaction				*0.054*				*0.056*
White British	9047	125	0.43	284.7 (239.0, 339.3)	White British	REF				REF			
South Asian	1256	11	0.03	293.4 (162.5, 529.7)	South Asian, 0% own ethnic density	0.79	0.30	,2.09	0.64	0.87	0.32	, 2.34	0.79
					South Asian, 90% own ethnic density	0.15	0.00	, 17.42	0.43	0.24	0.00	, 33.76	0.57
					p-value for interaction				*0.55*				*0.65*
White British	9047	125	0.44	284.7 (239.0, 339.3)	White British	REF				REF			
Irish	548	5	0.01	345.3 (143.7, 829.5)	Irish, 0% own ethnic density	0.19	0.02	, 2.10	0.18	0.19	0.02	, 2.17	0.18
					Irish, 11% own ethnic density	68.50	0.04	, 111556	0.26	53.86	0.02	,118227.8	0.31
					p-value for interaction				*0.22*				*0.26*

*Model 1: Adjusted for age and sex, with an interaction for ethnicity*own ethnic density; Model 2: Adjusted for age, sex, area-level deprivation, urbanicity, social fragmentation, diagnosis, marital status and substance use disorders and ethnicity*own ethnic density interaction; P values from Wald tests*

Supplementary Table S5: Sub-Hazard Ratios (sHRs) for all-cause mortality- sensitivity analysis[1]

	Total sample	Deaths	Model 1 sHR	Model 1 (95% CI)	Model 1 p value	Model 2 asHR	Model 2 (95% CI)	Model 2 p value
**Area level indicators**								
IMD (per increase in fifths; from less to more deprived)	-	-	1.05	1.01 ,1.09	0.02	0.98	0.94 ,1.02	0.32
Urbanicity (per increase in fifths; from less to more urban)	-	-	0.92	0.89 ,0.96	<0.001	0.96	0.92 ,1.01	0.11
Social fragmentation (per unit increase; from less to more fragmented)	-	-	0.93	0.89 ,0.97	0.0019	0.99	0.95 ,1.04	0.65
**Individual-level indicators**								
**Sex**								
Male	9610	908	REF	-	-	REF	-	-
Female	8591	859	1.17	1.06 ,1.29	0.0027	0.90	0.81 ,0.99	0.027
**Diagnosis**								
Non-affective	13160	1358	REF	-	-	REF	-	-
Affective	5041	409	0.91	0.81 ,1.02	0.10	0.83	0.74 ,0.93	0.0013
**Marital status**								
Married/ cohabiting	2781	267	REF	-	-	REF	-	-
Divorced/separated/widowed/single	15420	1500	0.90	0.78 ,1.03	0.13	1.15	1.01 ,1.32	0.041
**Substance use disorder**								
None	15046	1519	REF	-	-	REF	-	-
SUD	3155	248	0.75	0.66 ,0.86	<0.0001	1.07	0.94 ,1.22	0.29
**Interaction of ethnicity with areal ethnic density**								
**Lowest ethnic density area (0% ethnic minorities)**								
White British	-	-	REF			REF		
Ethnic minorities	-	-	0.65	0.49, 0.87	0.004	0.73	0.55, 0.97	0.029
**Highest ethnic density area (95% ethnic minorities)**								
White British	-	-	REF			REF		
Ethnic minorities	-	-	0.33	0.24, 0.45	<0.0001	0.45	0.33, 0.61	<0.0001
p-value for ethnicity X ethnic density interaction					*0.020*			*0.083*

Key: *-1767 deaths from all causes (total 18201 individuals with SMI); IMD: Index for Multiple Deprivation; Model 1 crude estimates; Model 2 adjusted for: age, interaction between area-level ethnic density X ethnicity, and all other displayed variables; p-values from Wald Tests; [1]Sensitivity analysis: sub-Hazard ratios (sHRs) for association of each variable with all-cause mortality with emigrations out of the cohort specified as a competing risk.*

Supplementary Figure S6: Scatter plots of own group density and total ethnic minority density

Figure S11a. Scatter plot of Black African own group density with total ethnic minority density (Black African N=2510; Total sample N=18201). Correlation coefficient 0.73

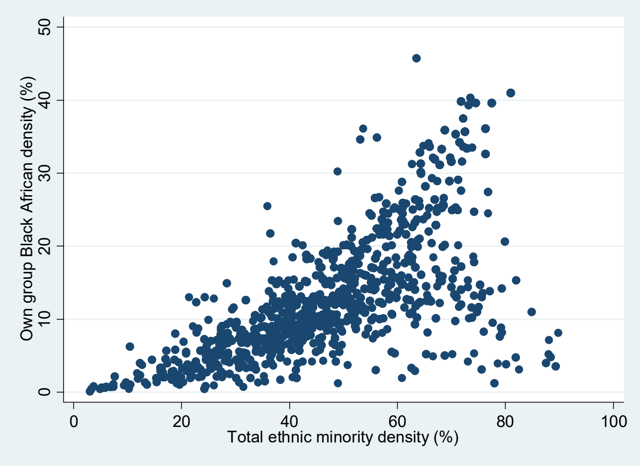

Figure S11b. Scatter plot of Black Caribbean own group density with total ethnic minority density (Black Caribbean N=4840; Total sample N=18201). Correlation coefficient 0.68

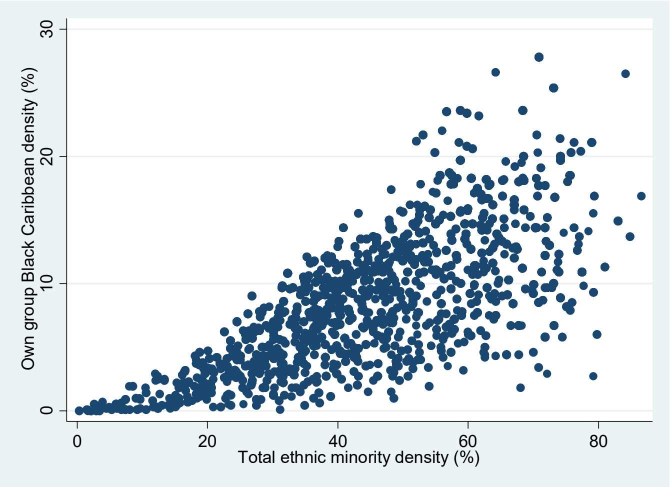

Figure S11c. Scatter plot of Irish own group density with total ethnic minority density (Irish N=548; Total sample N=18201). Correlation coefficient -0.31

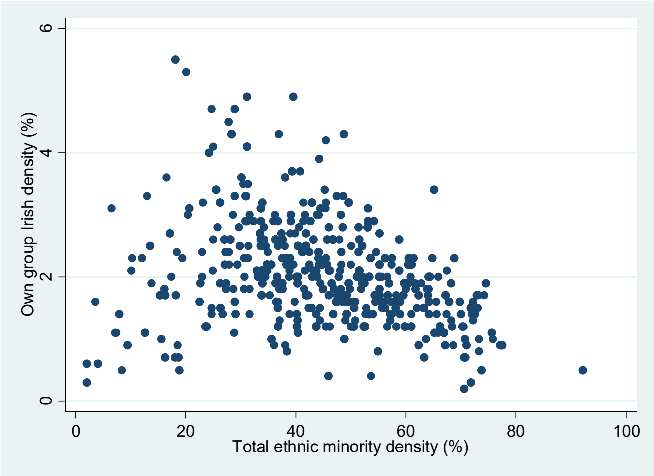

Figure S11d. Scatter plot of South Asian own group density with total ethnic minority density (South Asian N=1256; Total sample N=18201). Correlation coefficient 0.63.

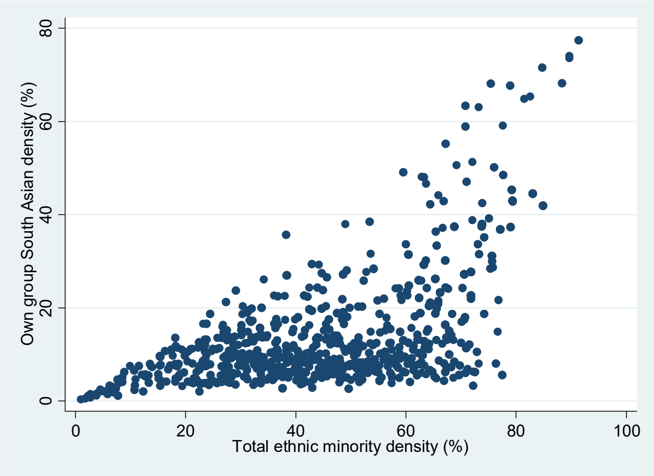

Supplementary Table S6: Search strategies and results for 'research in context' section

MeSH terms:

"Mental illness" AND

"Mortality" AND

Keyword search:

("residential characteristics" OR "neighbourhood characteristics" OR "multilevel analysis")

OR

§("ethnic density" OR "ethnic composition" OR "ethnic enclave*" OR "ethnic segregation" OR "ethnic concentration" OR "group concentration" OR "group density" OR "minority concentration" OR "minority density" OR "racial composition" OR "racial concentration" OR "racial homogeneity" OR "racial density" OR "racial segregation" OR "residential concentration" OR "residential segregation")

OR

("social fragmentation")

OR

("urban")

OR

("deprivation")

Search strategies for ethnic density terms were informed by a previous systematic review: "Shaw, R. J., Atkin, K., Bécares, L., Albor, C. B., Stafford, M., Kiernan, K. E., Nazroo, J. Y., Wilkinson, R. G. & Pickett, K. E. (2012). Impact of ethnic density on adult mental disorders: narrative review. British Journal of Psychiatry 201, 11-19".

Searches were supplemented by hand-searching the reference section of a recent systematic review "Hjorthoj C., Sturup A.E.,McGrath J.J., NordentoftM. Years of potential life lost and life expectancy in schizophrenia: a systematic review and meta-analysis. Lancet Psychiatry 2017; 4:295-301"

Supplementary Table S6, contd.: Retrieved studies for 'research in context' section

Study number	Study, author year	Region/ country	Study design	N, population	Measure(s)	Follow up period	Outcome(s)	Findings	Model adjustments/ confounders, notes
**Urbanicity**									
1	Fekadu et al 2015[1]	Ethiopia	Cohort	919 adults with Schizophrenia, bipolar disorders, severe depression, from 68378 screened	Rural/ Urban- not defined	10 years	All cause mortality	Adjusted models- Rural v. urban was HR: 1.15 (95% 0.73, 1.82)	Same cohort as study #2 but with longer follow up and broader diagnoses. Models adjusted for: Sex, age, marital status, educational status, age of onset, duration of illness, speed of onset, schizophrenia subtype, antipsychotics
2	Teferra et al 2011[2]	Ethiopia	Cohort	307 adults with schizophrenia from 68378 screened	Rural/ Urban/ semi-urban- although not defined	5 years	All-cause mortality	Adjusted models- Rural v. urban HR: 0.34 (95% CI: 0.14, 0.80)	Adjusted for sex, marital status, age, diagnosis, illness duration, illness severity and illness course
4.	Philips et al 2004[3]	China	Estimated from case-control study and survey data	19223 people in 1993 psychiatric survey data. Estimates based on 4·25 million people with schizophrenia in China.	Urban- people living in cities, rural- people living in either towns or in villages	n/a	Suicide	Rural vs. Urban (REF) residents with schizophrenia were more likely to commit suicide (RR 3.18 (95% CI: 1.96, 5.18), note rural vs. urban differences higher in	Sex stratified estimates

								men with schizophrenia (RR: 5.50 (95% CI: 2.03, 14.91) than in women with schizophrenia (RR: 2.28 (95% CI: 1.30, 4.01)	
6a.	Kisely et al 2005[4]	Nova Scotia, Canada	Cohort	221,048 patients in contact with psychiatric services or primary care. The sample includes other diagnoses aside from functional psychoses (eg, neuroses, dementia, alcohol disorders, mood disorders, personality disorders).	Average-derived household income by postcode in quartiles, measure for urbanicity (metropolitan Halifax') versus elsewhere in the province (non-metropolitan)	5 years	All-cause mortality	No difference by urbanicity (metropolitan area RR: 0.97 (95% CI: 0.92, 1.01) compared to non-metropolitan areas (reference)	Adjusted for diagnosis, sex, age, income, urbanicity and treatment setting
**Area-level deprivation**									
6b.	Kisely et al 2005[4]	-	-	-	-	-	-	Adjusted relative risk (RR) of mortality greater in people with mental disorders with increasing deprivation (RR: 1.24 (95% CI: 1.15, 1.33) in lowest deprivation category compared to least deprived category (reference).	Adjusted for diagnosis, sex, age, income, urbanicity and treatment setting

3	Tsai, 2014[5]	Taiwan	Cohort	60402 people with schizophrenia	1. Neighbourhood SES devised through taking average individual-level occupation and education<br>2. Urbanization (urban, suburban and rural)- defined through population density (cut points not given)	10 years	All cause mortality	Adjusted Hazard Ratios for lowest occupation neighbourhood compared to highest was 1.18 (95% CI: 1.09, 1.28). Adj. Hazard Ratio for lowest education neighbourhood compared to highest was 1.22 (95% CI: 1.11, 1.34).	Adjusted for age, sex, comorbidities, individual SES, level of urbanization and hospital characteristics
5.	Martin et al 2014[6]	Glasgow, Scotland	Cohort	4787 people with schizophrenia, 1784 with bipolar disorder, 67 with organic psychosis, 452 with psychotic depression, aged between 18-65 in Greater Glasgow	Scottish Index of Multiple Deprivation (SIMD)-domains include: current income, employment, health, education, access to services, crime and housing. Relative measure of deprivation.	4 years	All-cause and cause specific mortality (accidents, alcohol, cancer, cardiovascular, cerebrovascular, drugs, other, respiratory)	With increasing deprivation increasing mortality in both SMI and non-SMI groups. All-cause mortality rates higher in SMI group across fifths of deprivation compared to Scottish and local populations. Proportion of suicide higher in SMI in most and least affluent quintiles compared to local and national (Scotland) population.	Unadjusted mortality rates/ 10,000 population per 5 years
7.	Osborn et al, 2007[7]	UK	Cohort	General Practice Research Database (GPRD) with 46136 people with SMI and	Carstairs index of ward-level deprivation (derived from male unemployment,	Median follow up time 4.7 years (SMI group)	Suicide	Within full sample-adjusted Hazard Ratio (HR) for SMI compared to non-SMI was 10.52 (95% CI: 4.25, 26.05) in 20% least deprived and	Models adjusted for sex, age, calendar period, antidepressant prescription in previous six months and smoking status

				300426 without SMI	households with no car, overcrowding and head of household in unskilled manual work.	and 4.3 years (non-SMI group)		HR: 5.30 (95% CI: 2.77, 10.15) in 20% most deprived.<br><br>Within SMI population, suicide rate was lower with increasing deprivation. Adjusted Hazard Ratio for suicide mortality was 0.54 (0.40, 0.99) in most deprived fourth compared to the most affluent category (reference) of Carstairs deprivation index.	
**Material and social deprivation (the scale includes social fragmentation measures and measures for area-level deprivation)**									
8.	Vanasse et al 2012[8]	Quebec, Canada	Cohort	177850 people with a mood disorder diagnosis (bipolar disorders and depression) in 2006, followed for at least two years	Scale developed by Institut national de la santé publique du Québec based on 1. Proportion of people without certificate or high school diploma, 2. Proportion living alone; 3. Proportion separated, divorced or widowed; 4. Proportion single parent families. Ordered from least	Follow up for deaths within two years after diagnosis	All-cause mortality	Rate ratio comparing most materially/ socially deprived neighbourhoods with the least materially/ socially deprived neighbourhoods was women: RR 1.71 and men: 1.71, with a suggestion of a gradient underlying the association	Adjusted for age

	to most disadvantaged (fifths). The scale was partitioned to ‘social’ and ‘material’ deprivation.	

Key

Studies highlighted in red indicate that higher levels of urbanicity/ deprivation/ social fragmentation were associated with more adverse mortality outcomes in SMI

Studies highlighted in green indicate that higher levels of urbanicity/ deprivation/ social fragmentation were associated with less adverse mortality outcomes in SMI

Studies in black indicates no clear direction of association between area-level indicator and mortality outcome in SMI

References cited in table above

1. Fekadu, A., et al., Excess mortality in severe mental illness: 10-year population-based cohort study in rural Ethiopia. British Journal of Psychiatry, 2018. **206**(4): p. 289-296.
2. Teferra, S., et al., Five-year mortality in a cohort of people with schizophrenia in Ethiopia. BMC Psychiatry, 2011. **11**(1): p. 165.
3. Phillips, M.R., et al., Suicide and the unique prevalence pattern of schizophrenia in mainland China: a retrospective observational study. Lancet, 2004. **364**(9439): p. 1062-8.
4. Kisely, S., et al., Mortality in individuals who have had psychiatric treatment: Population-based study in Nova Scotia. British Journal of Psychiatry, 2005. **187**(6): p. 552-558.
5. Tsai, A.C., et al., Co-occurring epidemics, syndemics, and population health. The Lancet, 2017. **389**(10072): p. 978-982.
6. Martin, J.L., et al., Impact of socioeconomic deprivation on rate and cause of death in severe mental illness. BMC Psychiatry, 2014. **14**(1): p. 261.
7. Osborn, D., et al., Suicide and severe mental illnesses. Cohort study within the UK general practice research database. Schizophrenia Research, 2008. **99**(1-3): p. 134-8.
8. Vanasse, A., et al., [Health inequities in mood disorders based on material and social deprivation in dwelling sectors]. Canadian Journal of Psychiatry - Revue Canadienne de Psychiatrie, 2012. **57**(12): p. 772-81.
